# LncRNA UCA1 promotes keratinocyte-driven inflammation via suppressing METTL14 and activating the HIF-1α/NF-κB axis in psoriasis

**DOI:** 10.1038/s41419-023-05790-4

**Published:** 2023-04-20

**Authors:** Yibo Hu, Li Lei, Ling Jiang, Hongliang Zeng, Yushan Zhang, Chuhan Fu, Haoran Guo, Yumeng Dong, Yujie Ouyang, Xiaolin Zhang, Jinhua Huang, Qinghai Zeng, Jing Chen

**Affiliations:** 1 Department of Dermatology, the Third Xiangya Hospital, Central South University, No.138 Tongzipo Road, Changsha, Hunan 410013 PR China; 2 Center of Medical Laboratory Animal, Hunan Academy of Chinese Medicine, No.128 Yuehua Road, Changsha, Hunan 410013 PR China

**Keywords:** Gene silencing, RIG-I-like receptors, Psoriasis

## Abstract

Keratinocytes are closely associated with innate immunity and inflammatory responses, and are dysregulated during the development of psoriasis, but the underlying mechanisms are not yet fully understood. This work aims to reveal the effects of long non-coding RNA (lncRNA) UCA1 in psoriatic keratinocytes. UCA1 was identified as a psoriasis-related lncRNA that highly expressed in psoriatic lesions. The transcriptome and proteome data of keratinocyte cell line HaCaT showed that UCA1 could positively regulate inflammatory functions, such as response to cytokine. Furthermore, UCA1 silencing decreased inflammatory cytokine secretion and innate immunity gene expression in HaCaT, its culture supernatant also decreased the migration and tube formation ability of vascular endothelial cells (HUVECs). Mechanistically, UCA1 activated the NF-κB signaling pathway, which is regulated by HIF-1α and STAT3. We also observed a direct interaction between UCA1 and N6-methyladenosine (m^6^A) methyltransferase METTL14. Knocking down METTL14 counteracted the effects of UCA1 silencing, indicating that it can suppress inflammation. In addition, the levels of m^6^A-modified HIF-1α were decreased in psoriatic lesions, indicating that HIF-1α is a potential target of METTL14. Taken together, this work indicates that UCA1 positively regulates keratinocyte-driven inflammation and psoriasis development by binding to METTL14, and activating HIF-1α and NF-κB signaling pathway. Our findings provide new insights into the molecular mechanisms of keratinocyte-driven inflammation in psoriasis.

## Introduction

Psoriasis is a chronic inflammatory skin disease with a complex pathophysiological basis involving multiple cell types [1, 2]. Keratinocytes are the most abundant skin cells and mediate inflammatory response by secrete cytokines and chemokines such as interleukin 6 (IL6) and chemokine ligand 1 (CXCL1), and interact with other immune cells to enhance the inflammatory response in skin lesions [3]. These effects are caused by the activation of pattern recognition receptors (Toll-like receptors, TLR; nucleotide-binding oligomerization domain-like receptors, NLR), a kind of the innate immune response [4]. The role of keratinocytes in psoriasis has gained more attention in recent years, although the underlying mechanisms need further clarification.

Epigenetics is a non-inherited form of gene regulation [5–7]. Its influence has also been observed in several inflammatory skin diseases, including psoriasis [8–10], although their mechanisms are not yet fully understood. N6-methyladenosine (m^6^A) is the most common epigenetic modification of eukaryotic RNA [11]. Studies show that the m^6^A modification regulates embryonic development, stress response, tumor progression, and immunity [12–14]. In addition, there are reports of the involvement of m^6^A modification in psoriasis progression. For example, overall m^6^A modification levels are decreased in psoriatic lesions [8], the polymorphism of demethylase FTO has been associated with an increased risk of metabolic diseases in psoriasis patients [15]. The m^6^A is a dynamic modification regulated by methyltransferases such as METTL3 and METTL14, and demethylases, such as WTAP and FTO. In addition, the m^6^A residues are recognized by YTHDF1 and other readers, eventually affecting the transport, localization, translation, and degradation of downstream target RNAs [11]. However, the effects of m^6^A regulatory factors on immune function of psoriatic keratinocytes and the specific regulatory mechanisms remain unclear.

Urothelial cancer associated 1 (UCA1) is an long non-coding RNA (lncRNA) involved in the development of bladder cancer, gastric cancer, and colorectal cancer [16–18]. Recent studies show that UCA1 also has a regulatory role in inflammatory responses. For example, increased level of UCA1 in the blood is associated with an increased risk of acute respiratory distress syndrome in patients undergoing cardiopulmonary bypass [19], and UCA1 overexpression can worsen acute septic pneumonia by upregulating the expression of EZH2 and suppressing HOXA1 [20]. We previously found that the UCA1 is a functional regulator in skin cells, and is highly expressed in keratinocytes [21]. However, the potential regulatory role of UCA1 in keratinocyte-driven inflammation needs to be elucidated further.

In this study, we analyzed the transcriptome of psoriatic skin and the relationship between psoriasis development and UCA1. To evaluate its role in the inflammatory process, we manipulated UCA1 levels in keratinocytes, and performed functional assays. Furthermore, the role and regulatory mechanisms of METTL14 in UCA1-mediated keratinocyte-driven inflammation was also explored.

## Results

### The role of UCA1 in psoriasis development

**Fig. 1:**
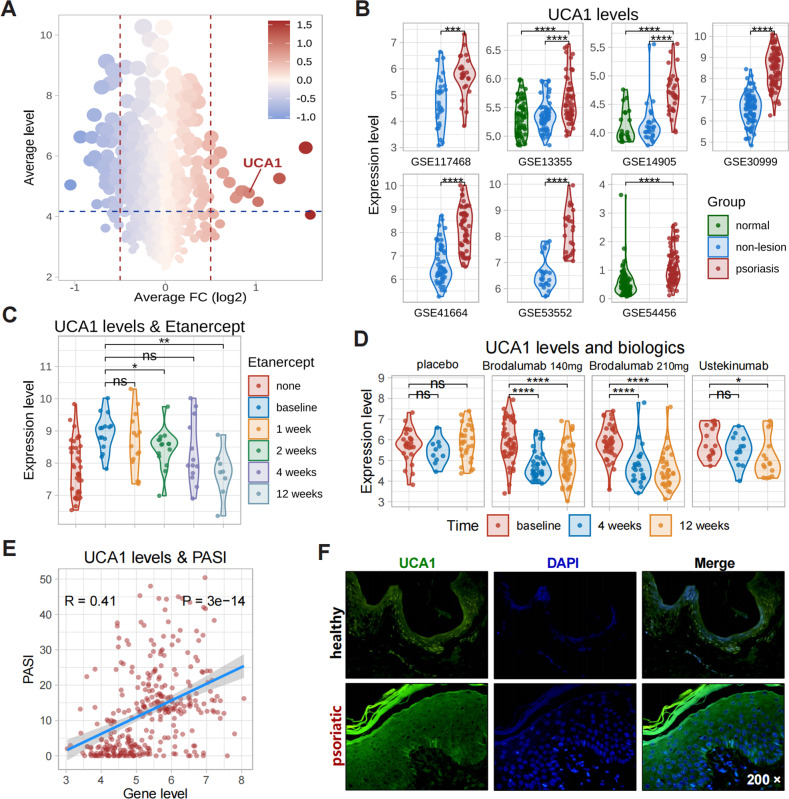
The role of UCA1 in psoriasis. **A** The average foldchange of lncRNA expression levels in integrated GEO transcriptome datasets. **B** UCA1 levels in different psoriasis datasets. **C**, **D** UCA1 levels in psoriatic lesions treated with different biologics (GSE41664 and GSE117468). **E** The correlation (Pearson coefficient) between UCA1 expression and PASI in psoriatic lesions (*n* = 323, GSE117468). **F** Representative fluorescence in situ hybridization (FISH) images of UCA1 in 40 psoriatic and 20 normal skin specimens (magnification: 200×).

We analyzed nine transcriptome datasets of psoriatic skin from the Gene Expression Omnibus (GEO) database (Table. S1). Gene set enrichment analysis (GSEA) revealed significant activation of inflammation-related functions and signaling pathways, including cytokine activity, Janus kinase/signal transducer and activator of transcription (JAK/STAT), and nuclear factor kappa B (NF-κB), in the psoriatic lesions (Figure. S1). In addition, 1176 lncRNAs were identified among the differentially expressed genes (DEGs) between psoriatic and non-lesional samples (Figure. S2A-B), and UCA1 was one of the top 10 up-regulated lncRNAs in the psoriatic lesions (Figure. S2C-D, Fig. 1A-B). In GSE41664 [22] and GSE117468 [23] datasets, treatments with biologics (Etanercept, Brodalumab, and Ustekinumab) deceased the excessive UCA1 expression (Fig. 1C-D). Furthermore, UCA1 expression was positively correlated with psoriasis area and severity index (PASI; *R* = 0.41, *P* < 0.05; Fig. 1E). The bioinformatics results were confirmed by detecting UCA1 levels in specimens of psoriatic lesions and healthy skin (Fig. 1F, Figure. S2E-F). Taken together, UCA1 is likely involved in psoriasis development and may regulate the keratinocyte-driven inflammatory response.

### UCA1 promotes inflammatory functions in psoriatic keratinocytes

**Fig. 2:**
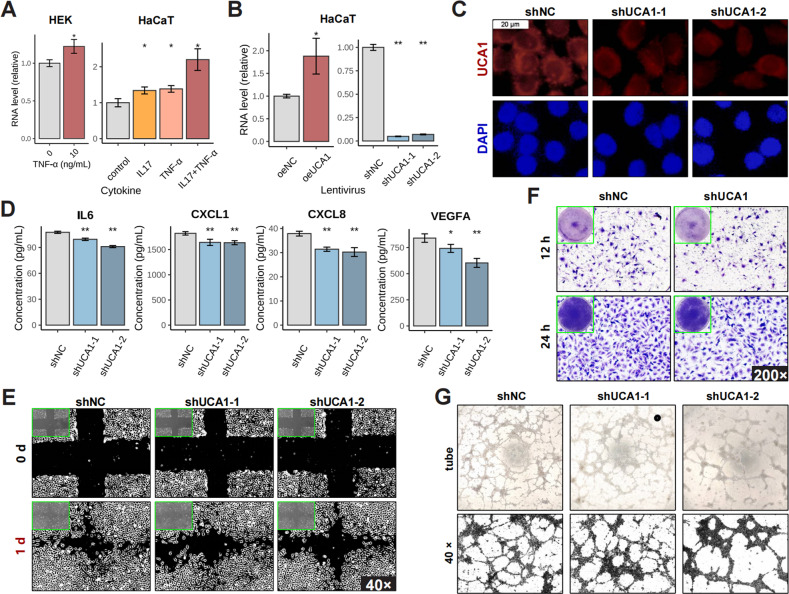
The functions of UCA1 in psoriatic keratinocytes. **A** UCA1 levels in HEK and HaCaT cells stimulated with IL17/TNF-α (both 10 ng/mL). **B** HaCaT cells were transfected with lentivirus (oeUCA1: UCA1 over-expression; shUCA1-1/2: two short hairpin RNAs used in UCA1 knocking down). **C** Representative FISH images showing in situ UCA1 expression (white scale bar: 20 μm). **D** The levels of cytokines and chemokines in the culture supernatant of HaCaT cells. **E**, **F** The migration ability of HUVEC cultured with the medium supernatant of HaCaT cells was evaluated by wound healing assay (magnification: 40×) and Transwell assay (magnification: 200×). **G** Tube formation assay of HUVECs cultured with the medium supernatant of HaCaT cells in Matrigel (magnification: 40×). (*P < 0.05, **P < 0.01; error bars represent standard deviations).

We had previously demonstrated that UCA1 is highly expressed in keratinocytes [21]. Consistent with this, UCA1 was markedly upregulated in primary human epidermal keratinocytes (HEK) and immortalized human keratinocytes HaCaT stimulated with IL17 and/or tumor necrosis factor alpha (TNF-α) (Fig. 2A), which was accompanied by a psoriasis-like inflammatory response (Figure. S3A-G). To further explore the role of UCA1 in keratinocytes, we respectively overexpressed (oeUCA1) and knocked down (shUCA1) UCA1 in inflammatory HaCaT cells (Fig. 2B-C). In GEO datasets, UCA1 expression was positively correlated with that of inflammatory genes such as IL6 and CXCL1 (Figure. S4A). Similarly, UCA1 knockdown significantly reduced the levels of IL6, CXCL1, CXCL8, and vascular endothelial growth factor A (VEGFA) mRNAs in HaCaT cells (Figure. S4B), as well as the secreted protein levels in the culture supernatant (Fig. 2D). Since IL6 and VEGFA are involved in psoriatic angiogenesis [24], we cultured human umbilical vein endothelial cells (HUVEC) with the medium supernatant of UCA1-knockdown HaCaT cells, and observed a significant inhibition in the migration (Fig. 2E-F) and tube formation (Fig. 2G) of HUVECs.

**Fig. 3:**
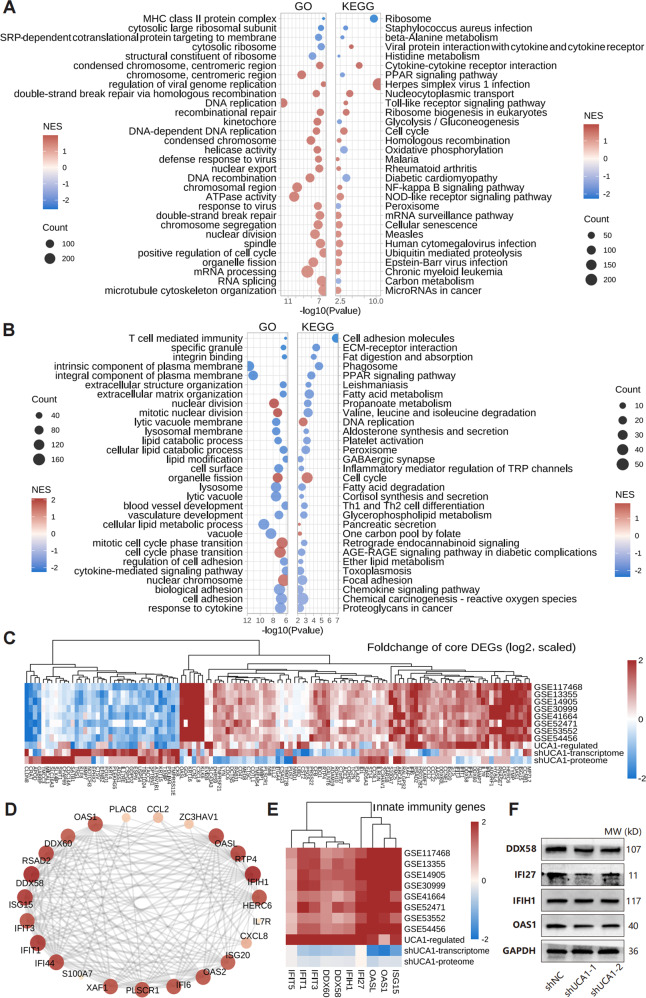
The transcriptome and proteome data of HaCaT cells. **A** The transcriptome data of HaCaT cells with UCA1 over-expression and knockdown were obtained using DNB-seq high throughput sequencing, and the foldchange of gene expression was weighted (log_2_FC_weighted_ = log_2_FC_oe_ - log_2_FC_sh_). The enriched functions and signaling pathways were identified by GSEA. **B** The proteome data of UCA1-knockdown HaCaT cells was obtained using isobaric tags for relative and absolute quantification (iTRAQ), and functionally annotated by GSEA. **C** The transcriptome and proteome data of HaCaT and eight GEO datasets were integrated by Robust rank aggregation to screen out core DEGs. **D** Hub genes were presented using a PPI network. **E** The expression regulation of hub genes in GEO datasets and HaCaT. **F** The protein levels of innate immunity genes in UCA1-knockdown HaCaT cells.

To further explore the underlying mechanisms, we analyzed the transcriptome data of UCA1-overexpressing and UCA1-knockdown HaCaT cells, and calculated the foldchange of the expression of genes relative to the controls (log_2_FC_weighted_ = log_2_FC_oe_ - log_2_FC_sh_; Figure. S4C-D). In addition, the proteome data of UCA1-knockdown HaCaT cells was also analyzed (Figure. S4E-F). GSEA results of the transcriptome data suggested that UCA1 promotes DNA replication, mRNA processing, response to virus, cytokine-cytokine receptor interaction, etc. (Fig. 3A, Figure. S5). According to the proteome data, UCA1 knockdown inhibited vascular development, response to cytokine, cell adhesion, fatty acid metabolism, chemokine, etc. (Fig. 3B, Figure. S6). Subsequently, we identified 121 core DEGs from the transcriptome and proteome data of HaCaT cells and eight GEO datasets (Fig. 3C). Protein-protein interaction (PPI) network analysis indicated that most hub genes are related to innate immunity (Fig. 3D). Ten representative genes (DDX58, DDX60, IFI27, IFIH1, IFIT1, IFIT3, IFIT5, ISG15, OAS1, and OASL) were differentially expressed in multiple datasets (Fig. 3E, Figure. S7A). Furthermore, DDX58, IFI27, and OAS1 were induced by IL17/TNF-α stimulation in HaCaT cells (Figure. S7B-C), but suppressed by UCA1 knockdown (Figure. S7D, Fig. 3F). These results confirm that UCA1 promotes the keratinocyte-driven inflammatory response, possibly via modulation of the innate immune system.

### UCA1 activates NF-κB signaling pathway and HIF-1α/STAT3

**Fig. 4:**
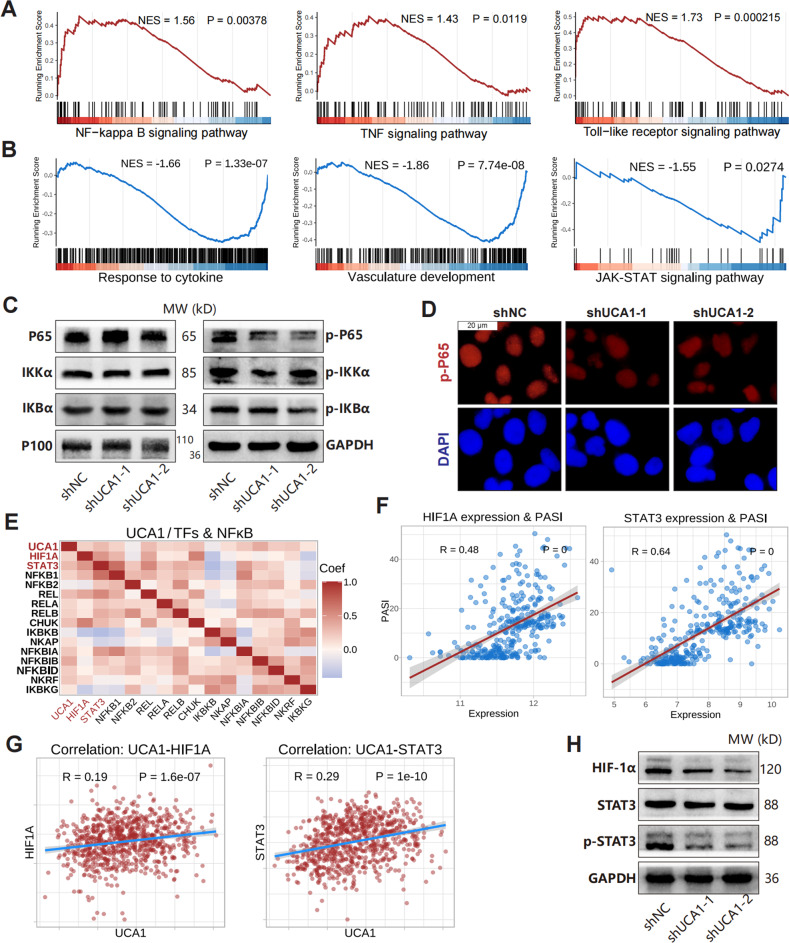
The inflammatory pathways and transcription factors regulated by UCA1. **A** Upregulated signaling pathways in HaCaT transcriptome data according to GSEA using log_2_FC_weight_. **B** Down-regulated functions in proteome data of UCA1-knockdown HaCaT cells. **C**, **D** The levels of total and phosphorylated NF-κB proteins were measured by western blotting and immunofluorescence (white scale bar: 20 μm). **E** Correlation (Pearson coefficient) between NF-κB genes and UCA1 expression in psoriatic lesions. **F** Correlation between HIF-1α and STAT3 expression with PASI (*n* = 323, GSE117468). **G** Correlation between HIF-1α, STAT3, and UCA1 expression in psoriatic lesions. H. The levels of total HIF-1α and p-STAT3 proteins in UCA1-knockdown HaCaT cells.

Transcriptome data of HaCaT cells showed that UCA1 likely activates the NF-κB, TNF, and TLR signaling pathways (Fig. 4A). In addition, the proteome data also confirmed that UCA1-knockdown suppressed the response to cytokine, vasculature development, and the JAK/STAT signaling pathway (Fig. 4B). In GEO datasets, STAT1, STAT3, NFKB1, REL, and RELB mRNAs were upregulated in psoriatic lesions, whereas AKT2/3 was downregulated (Figure. S8A-C). Furthermore, UCA1 knockdown decreased the levels of phosphorylated NF-κB proteins (P65, IKKα, and IKBα) but did not affected the total protein levels in HaCaT (Fig. 4C-D). Thus, these findings suggest that UCA1 may promote the inflammatory response via the NF-κB signaling pathway.

HIF-1α and STAT3 are critical transcription factors involved in inflammation, and were upregulated in psoriatic lesions and decreased under biologics treatments in GEO datasets (Figure. S9A-B). In addition, HIF1A and STAT3 expression levels were positively correlated to NF-κB genes (Fig. 4E), PASI scores (Fig. 4F), and UCA1 expression (Fig. 4G). Moreover, knocking down UCA1 in HaCaT cells also downregulated HIF-1α and phosphorylated STAT3 proteins (Fig. 4H). These findings suggest that HIF-1α and STAT3 are potential targets of UCA1 and may activate the inflammatory functions in keratinocytes.

### UCA1 specifically binds to methyltransferase METTL14

**Fig. 5:**
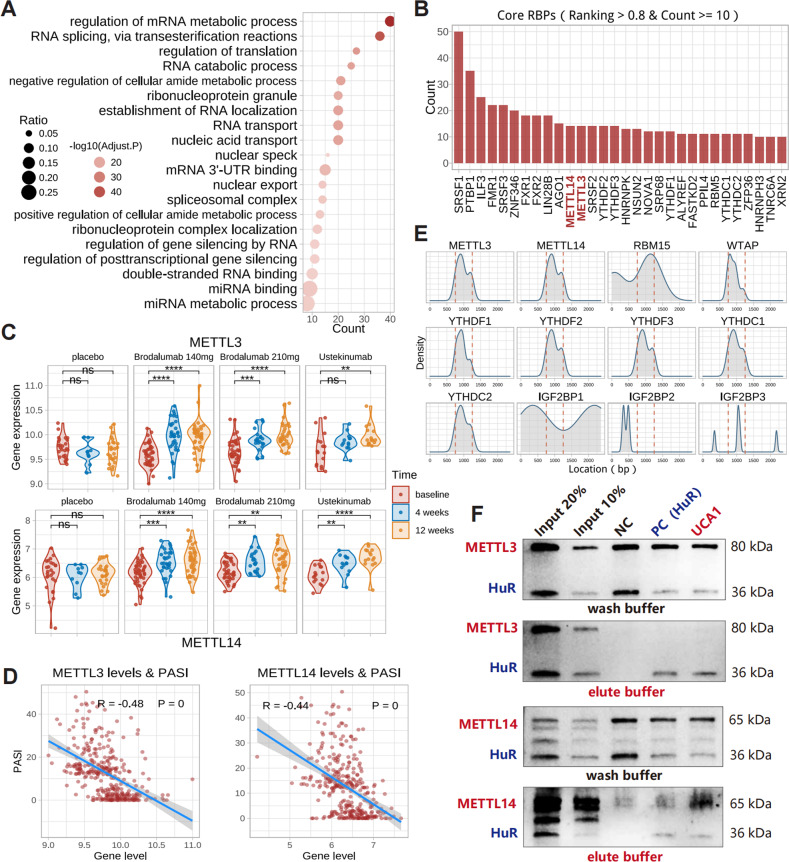
UCA1-binding proteins. **A** Functions of UCA1-binding proteins (predicted by catRAPID: http://service.tartaglialab.com/page/catrapid_group; ranking score >0.8). **B** Thirty top-ranked UCA1-binding proteins (ranking score > 0.8 & binding sites ≥10). **C** METTL3 and METTL14 levels in psoriatic lesions treated with biologics (GSE117468). **D** Correlation between METTL3 and METTL14 expression and PASI (*n* = 323, GSE117468). **E** The distribution of protein-binding sites on UCA1 RNA. **F** UCA1-binding proteins in HaCaT were enriched by biotin-labeled UCA1 probes (706 to 1,285 bases of UCA1) and identified by western blotting.

UCA1 was mainly distributed in the cytoplasm and peri-nuclear regions of HaCaT cells (Fig. 2C). Analyses of catRAPID-predicted results revealed that UCA1-binding proteins (ranking score > 0.8) mainly involve in RNA processing and transport (Fig. 5A). Furthermore, 30 of them have more than 10 UCA1-binding sites (Fig. 5B), including m^6^A-related gene METTL3, METTL14, YTHDF1/2/3, and YTHDC1/2. We then analyzed the methylated RNA immunoprecipitation sequencing (MeRIP) dataset (GSE155702) and found decreased m^6^A-enriched peaks in psoriatic lesions (Figure. S10A-B). In addition, METTL3 and METTL14 were down-regulated in psoriasis (Figure. S10C) and restored after biologics treatments (Fig. 5C). Moreover, both METTL3 and METTL14 expression were negatively correlated with PASI (Fig. 5D), inflammatory genes, and innate immunity genes in the psoriatic lesions (Figure. S10D-E). The predicted sites of m^6^A proteins binding to were mainly distributed across 750-1,250 bp region of the UCA1 RNA (Fig. 5E). RNA pull-down assay further confirmed that UCA1 specifically binds to METTL14 but not METTL3 (Fig. 5F). Taken together, METTL14 is a target of UCA1 and may be involved in UCA1-induced inflammation.

### METTL14 involves in UCA1-induced keratinocyte-driven inflammation

**Fig. 6:**
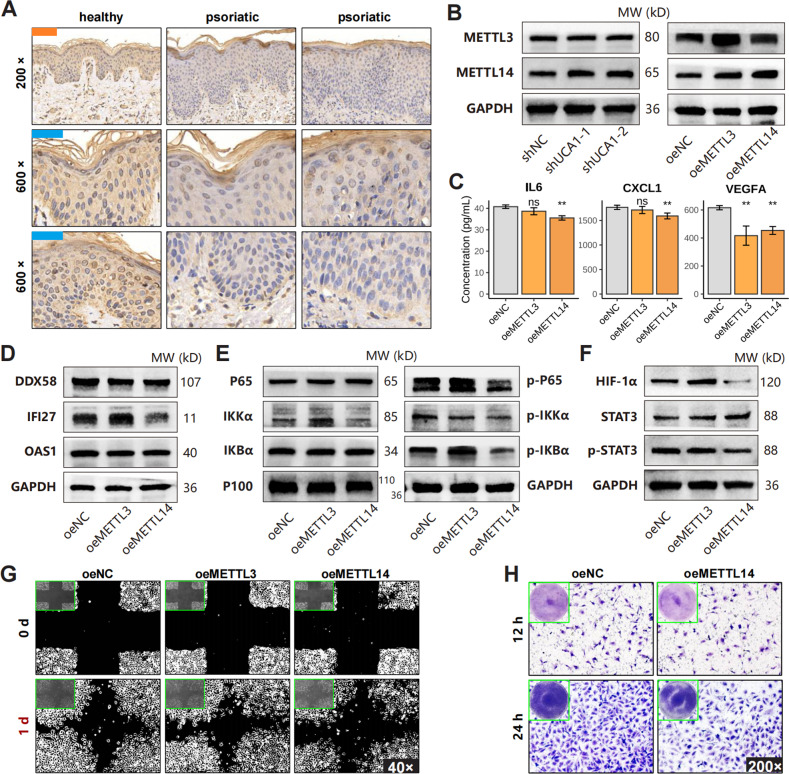
The function of METTL14 in psoriasis. **A** Representative images showing METTL14 expression in psoriatic and normal skin specimens (20 normal skin, 40 psoriatic lesions). **B** METTL3 and METTL14 protein levels in HaCaT cells transfected with the expression plasmids respectively (oeMETTL3, oeMETTL14). **C** Levels of indicated cytokines in the medium supernatants of METTL3/4-overexpressing HaCaT cells. **D**–**F** The levels of indicated proteins in HaCaT cells. **G**–**H** The migration ability of HUVEC cultured with HaCaT medium supernatant. (*P < 0.05, **P < 0.01; error bars represent standard deviations).

METTL14 was downregulated in psoriatic skin and inflammatory HaCaT cells (Fig. 6A, Figure. S11), and was restored following UCA1 knockdown (Fig. 6B). Furthermore, METTL14 overexpression in HaCaT cells (Figure. S12A-C) significantly decreased the levels of secreted inflammatory (IL6, CXCL1, VEGFA) and innate immunity-related (DDX58, IFI27) proteins (Fig. 6C-D), and also suppressed the NF-κB signaling pathway, HIF-1α, and p-STAT3 proteins (Fig. 6E-F). Consistent with this, HUVECs grown in the medium supernatant of METTL14-overexpressing HaCaT cells showed poor migration (Fig. 6G-H).

**Fig. 7:**
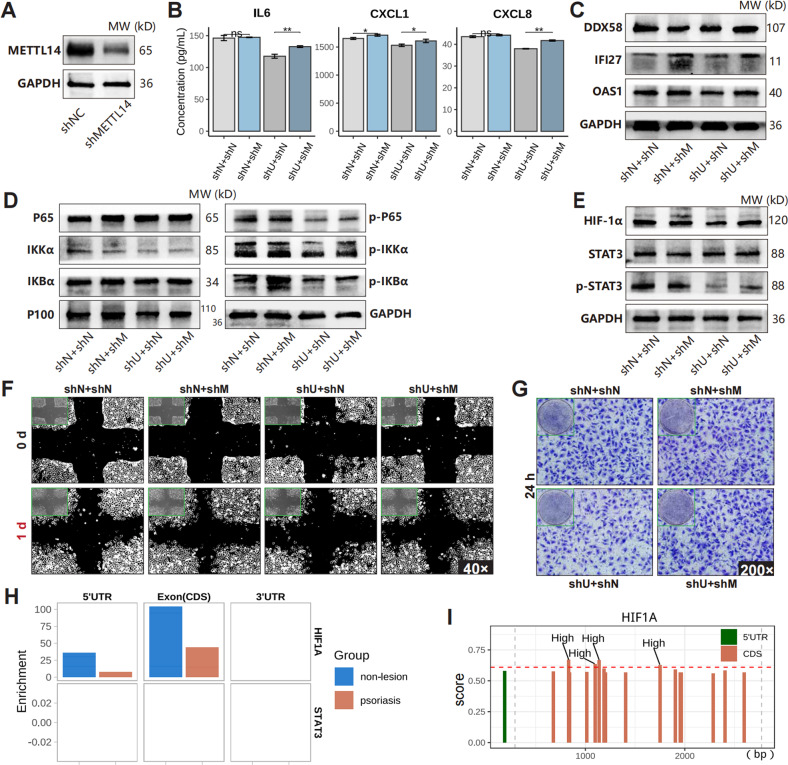
The functions of METTL14 in keratinocyte-driven inflammation. **A** METTL14 was knocked down by short hairpin RNA and lentivirus transfection. **B** The concentrations of secreted proteins in HaCaT culture supernatant (shN: knockdown control, shU: UCA1 knockdown, shM: METTL14 knockdown). **C**–**E** The levels of indicated proteins in HaCaT cells. **F**–**G** The migration ability of HUVEC treated with HaCaT culture supernatant. **H** The m^6^A levels of HIF1A and STAT3 in psoriatic skin (GSE155702). **I** The m6A modification sites of HIF1A RNA were predicted by SRAMP (http://www.cuilab.cn/sramp/). (*P < 0.05, **P < 0.01; error bars represent standard deviations).

On the other hand, silencing METTL14 (Fig. 7A, Figure. S13A-B) in the UCA1-knockdown HaCaT cells restored the levels of secreted cytokines and chemokines (IL6, CXCL1, CXCL8) and innate immunity genes (DDX58, IFI27, OAS1) (Fig. 7B-C), activated the NF-κB signaling pathway, and upregulated HIF-1α and p-STAT3 (Fig. 7D-E). Consequently, the migration ability of HUVEC was partially restored after METTL14 knockdown due to the increased levels of cytokines in the medium supernatant of HaCaT cells (Fig. 7F-G). Moreover, MeRIP data (GSE155702) indicated that the m^6^A levels of HIF1A RNA were lower in psoriatic lesions (Fig. 7H). Potential m^6^A modification sites predicted by SRAMP were around 1,000^th^ base of HIF1A RNA (Fig. 7I). Therefore, our results suggest that UCA1 can bind to METTL14 protein and further induce HIF-1α expression and inflammatory response in psoriatic keratinocytes.

## Discussion

Our findings suggest that lncRNA UCA1 promotes keratinocyte-driven inflammation in psoriasis by targeting and inhibiting METTL14 protein and then activating the HIF-1α/STAT3 and NF-κB signaling pathways. Furthermore, the expression levels of both UCA1 and METTL14 were associated with the severity of skin lesions, and had opposing effects on the inflammatory response. Thus, the UCA1-METTL14 axis plays a key role in psoriatic keratinocytes.

Additionally, keratinocytes are involved in antigen presentation and innate immune response [25]. Consistent with this, our results indicated that the activation of innate immunity in keratinocytes plays an important role in psoriatic inflammation. Recent studies show that RIG-1 (also known as DDX58) and IFI27 are key initiating factors in psoriatic inflammation [26, 27], although the exact mechanisms of their activation remain unclear. In addition, transcriptomics, proteomics and experimental data showed a positive relationship between UCA1, innate immunity gene expression, and inflammatory response, which suggests that UCA1 is a potential upstream regulator of DDX58 and IFI27.

We found that UCA1 was significantly overexpressed in psoriatic lesions and activated the inflammatory pathway in keratinocytes. However, Ma et al. reported that UCA1 acts as a suppressor of NF-κB activity in psoriatic inflammation, which is inconsistent with our findings [28]. These differences may be due to several factors. First, qPCR assay typically uses glyceraldehyde-3-phosphate dehydrogenase (GAPDH) as an internal control; however, GAPDH expression and aerobic glycolysis are increased in psoriatic lesions [29, 30], which can affect the accuracy of tissue detection results. Furthermore, cytokine-stimulated HaCaT cells are commonly used to simulate psoriasis in vitro, and there is evidence that psoriatic inflammation depends on the synergistic effect of multiple cytokines, particularly IL17A [31, 32]. In addition, different experimental models have their own distinct limitations [33], and treatments may produce disparate inflammatory effects, thus influencing UCA1 function. Nevertheless, since UCA1 has been implicated in several inflammatory diseases such as acute respiratory distress syndrome [19], sepsis [34], pneumonia [20], and acute ischemic stroke [35], we can surmise that UCA1 plays a pro-inflammatory role in psoriasis.

METTL14 can regulate the inflammatory response in vascular endothelial cells and renal podocytes by targeting SIRT1 and FOXO1 [36, 37], and modulates TNF-α-induced gene expression in mesenchymal stem cells [38]. This study is the first to show that METTL14 is involved in psoriatic inflammation. According to MeRIP data (GSE155702) [8], the m^6^A levels were slightly decreased in psoriatic lesions, which is consistent with the down-regulation of METTL14 expressions. Our findings indicate that METTL14 inhibits the inflammatory response in psoriatic keratinocytes. Moreover, m^6^A also regulates the genes involved in virus defense and innate immunity. For example, DDX5 was found to interact with METTL3 and increase the m^6^A modifications of p65 RNA, which facilitates its YTHDF2-mediated degradation and inactivates the NF-κB signaling pathway [39]. In addition, METTL14-induced m^6^A modification can destabilize the RNAs of mitochondrial antiviral signaling proteins, which inhibits downstream interferon-β production and response to RNA viruses [40]. This suggests that METTL14 may also regulate innate immunity in psoriasis.

The m^6^A modifications of lncRNAs can affect their expression and functions [41, 42], although little is known regarding the effect of lncRNAs on m^6^A-related genes. Our findings indicate that UCA1 can specifically bind to and suppress the METTL14 protein. In contrast, METTL14 knockdown can partially restored the impaired NF-κB signaling pathway activation and inflammatory response in UCA1-knockdown keratinocytes, suggesting that METTL14 is regulated by UCA1. HIF-1α is known to interact with NF-κB and UCA1 [43, 44], and was overexpressed in psoriatic lesions. Furthermore, the m^6^A levels of HIF-1α RNA was decreased in the lesions, which suggests that HIF-1α might be a target of METTL14.

Our study has some limitations that ought to be considered. Though we detected high-quality m^6^A modification sites on HIF-1α RNA, there was no direct evidence to confirm that UCA1 binding to METTL14 leads to reduced m^6^A modification and increased expression of HIF-1α. In addition, immune cells are the essential effectors of inflammation response in psoriasis, whereas we focused only on the functions of keratinocytes and vascular endothelial cells. These limitations will be addressed in subsequent studies in the future.

## Conclusions

The lncRNA UCA1 is upregulated in psoriatic lesions and promotes the keratinocyte-driven inflammatory response. It binds to the m^6^A methyltransferase METTL14 and upregulates HIF-1α, and the NF-κB signaling pathway in keratinocytes. Our findings provide novel insights into the molecular mechanisms underlying psoriatic inflammation, and demonstrate the diagnostic and therapeutic potential of UCA1 and METTL14.

## Materials and methods

### Experimental design

HEK and HaCaT were used to observe the inflammatory response and molecular mechanisms. HUVEC was used to observe the effects of keratinocyte-secreted cytokines on angiogenesis, and 20 normal skin and 40 psoriatic lesion specimens were collected to validate the UCA1 and METTL14 expression.

### Cell culture

HEK was isolated from circumcised foreskins of adolescent volunteers as described previously [45, 46] and cultured in Keratinocyte Growth Medium 2 supplemented SupplementPack (Promo Cell, Germany). The HaCaT cells and HUVECs were purchased from Procell (Wuhan, China) and cultured in Roswell Park Memorial Institute 1640 (RPMI-1640) and Dulbecco’s modified Eagle’s medium (DMEM) supplemented with 10% fetal bovine serum (FBS) and 1% penicillin-streptomycin solution. To model psoriasis, cells were stimulated with recombinant human IL17 (Sino Biological, China) and/or TNF-α (Proteintech, USA) in serum-free medium for 24 h. To prepare HaCaT culture supernatant for culturing HUVECs, half of the medium was replaced with fresh serum-free medium after 16 h of cytokine treatments. The supernatant was collected 8 h later and centrifuged at 3,000 rpm for 10 min.

### Cell transfection

The sequences of UCA1, METTL3, and METTL14 genes were referenced from the NCBI human genome database (GRCh38/hg38). The coding sequences (CDS) of METTL3 and METTL14 were synthesized by GenePharma (Suzhou, China) and cloned in pcDNA3.1 plasmid vectors. Short hairpin RNAs (shRNAs) were synthesized by GENE Chem (Shanghai, China), GenePharma and HANBIO (Shanghai, China) and packaged with lentivirus. The cells were seeded in 12-well plates, and infected with 5-20 μL lentivirus per well according to the multiplicity of infection (MOI). The transfected were screened using puromycin (1-5 μg/mL) or blasticidin (1–10 μg/mL) one week later. Plasmids were transfected using Lipofectamine 3000 (Thermo Fisher, USA) according to the manufacturer’s instructions. The medium was replaced 16 h later, and the cells or supernatant was collected 1-2 days after transfection.

### Transcriptome data

Part of the data was retrieved from the GSE117468 [23], GSE13355 [47], GSE14905 [48], GSE30999 [49], GSE41664 [22], GSE52471 [50], GSE53552 [51], GSE54456 [52], and GSE155702 [8], which include eight transcriptome datasets and one MeRIP (m^6^A epigenomics) dataset, from GEO database. Detailed information is provided in the supplementary material. In addition, HaCaT cells were transfected with lentiviruses as specified (oeNC: over-expression control, oeUCA1: UCA1 over-expression, shNC: knockdown control, shUCA1-1: UCA1 knockdown 1, shUCA1-2: UCA1 knockdown 2), with each group containing one sample. Total RNA was extracted from 10^7^ cells and the cDNA libraries were sequenced using the DNBSEQ platform, Huada BGI (Shenzhen, China). Finally, a total of 20,928 known genes were detected.

### Proteome data

HaCaT cells were transfected with lentiviruses (shNC: knockdown control; shUCA1: UCA1 knockdown) and each group contained three replicates. The total RNA and protein were extracted from 10^7^ cells. Huada BGI was hired to establish cDNA libraries and process proteins. The iTRAQ technique was used to obtain proteome data. A total of 921,604 secondary spectrum diagrams were generated, and 7,055 proteins were identified using the 1% false discovery rate (FDR) criterion. Proteome data were quantified using IQuant (Huada BGI, China), and the change in protein levels in shUCA1 group was calculated relative to that of shNC group. Concurrently, transcriptome data was obtained via the DNBSEQ platform, and a total of 16,409 known genes were detected.

### Bioinformatics analysis

Transcriptome and proteome data were analyzed using Ubuntu 20.04 LTS (Focal Fossa) system and R software (version 4.0.5). Core R packages included tidyverse, reshape2, Hmisc, ggpurb, ggplot2, pheatmap, GSVA, clusterProfiler, enrich plot, limma, and Deseq2. The raw data (CEL files) of the microarray were processed using the package affy, and the gene expression matrix was log_2_-converted. The expression matrix of GSE54456 was converted to reads per kilobase per million (RPKM) unit; the transcriptome data of HaCaT was also converted to fragments per kilobase of exon model per million reads mapped (FPKM) unit. GSEA, Robust rank aggregation, Pearson correlation analysis, and PPI network analysis were performed.

### Tube formation assay

The plates and pipettes were pre-cooled at −20 °C. High concentration Matrigel (Corning, USA) was melted at 4 °C, and 100 μL aliquots were dispensed into each well of 48-well plates. Once the Matrigel solidified in the incubator, 100 μL HUVEC suspension was seeded at the density of 2–4 × 10^3^ cells/well, and 100 μL keratinocyte culture supernatant was added (1:1, v/v). The cells were then incubated for 2-12 h and observed at 1 h intervals under the microscope (Olympus, Japan) for tube formation.

### Wound healing assay

The HUVECs were cultured in a 6-well plate until 100% confluent, and the monolayer was scratched longitudinally and transversely using a 200 μL pipette tip. After rinsing off the dislodged cells, serum-free medium and keratinocyte culture supernatant (1:1, v/v) was added to the wells. The wound area was observed at 0, 24, and 48 h to monitor healing. The images were processed using Image J and color-reversed to make the scratches look sharper.

### Transwell assay

The HUVECs were seeded in the upper chambers of a transwell insert in a 24-well plate at the density of 1-2 × 10^4^ cells/well in 200 μL serum-free medium and 200 μL keratinocyte culture supernatant. The lower chambers were filled with 500 μL complete medium. After incubating for 12 and 24 h, the cells that migrated to the bottom (outer) of the membrane were counted.

### RT-PCR

Total RNA of cells was extracted using a Fast200 RNA extraction kit (Fastagen, China), and 1 μg RNA per sample was reversed transcribed into cDNA. The relative gene expression levels were measured by quantitative real-time polymerase chain reaction (qPCR). Primers were designed using Primer-Blast.

### Western blotting (WB)

Total protein of cells was extracted using RIPA buffer supplemented with protease and phosphatase inhibitor cocktails (Roche, Switzerland). Equal amounts of protein per sample were separated by SDS-PAGE and transferred to membranes for western blotting. After blocking with 5% bovine serum albumin (BSA) at room temperature for 1-2 h, the blots were incubated overnight with primary antibodies (1:500–2000, v/v) at 4 °C, washed with tris buffered saline-Tween (TBS-T), and then incubated with secondary antibodies (1:5000–20,000, v/v) for 1 h at room temperature. Protein bands were visualized using enhanced chemiluminescence reagents.

### Immunofluorescence (IF)

The cells were seeded in 24-well plates, cultured till 30–50% confluence, then fixed with 4% paraformaldehyde and permeabilized with 0.5% Triton X-100 for 30 min. After blocking with 5% BSA at room temperature for 1-2 h, the cells were incubated overnight with primary antibodies (1:100–400, v/v) at 4 °C, washed with TBS-T and incubation with fluorescein-labeled secondary antibodies (1:1000, v/v) for 1 h at room temperature away from light. The cells were counterstained with DAPI (1:1000, v/v) and observed under a fluorescence microscope.

### Enzyme-linked immunosorbent assay (ELISA)

The cells were lysed by freezing and thawing five times, and the lysates were centrifuged at 3000 rpm for 10 min. The levels of cytokines and chemokines were measured using specific ELISA kits from Elabscience (Wuhan, China) and JINGMEI (Yancheng, China). The absorbance was measured at 450 nm using a multimode reader (PerkinElmer, USA) within 15 min.

### Immunohistochemistry (IHC)

IHC was performed using the Pv-9000 universal two-step detection kit (ZSGB-BIO, China). Normal skin and psoriatic lesion specimens were embedded in paraffin and cut into thin sections (3 μm). Following dewaxing and hydration, the sections were heated in citrate buffer (pH 6) in a microwave, and then treated with hydrogen peroxide to quench endogenous peroxidase. After blocking non-specific binding, the sections were incubated overnight with primary antibodies (1:50–400, v/v) at 4 °C, washed with PBS and then sequentially with the enhancement solution and enzyme-labeled IgG polymer for 20 min at 37 °C. The staining was developed using 3,3′-diaminobenzidine (DAB) for 30–100 s at room temperature in the dark and the slides were washed once the tissues turned brown-yellow under a microscope.

### Fluorescence in situ hybridization (FISH)

Tissue and cellular FISH Kits and FISH probe mixture for tissue or intracellular UCA1 detection was purchased from GenePharma, and RIBOBIO (Guangzhou, China). After blocking and pre-hybridization according to the kit specification, the tissue sections or cells were incubated overnight with 100 μL UCA1 probe working solution (1:50, v/v) at 37 °C in the dark and then washed with SSC buffer (4×, 2×, 1×) at 43 °C.

### RNA pull-down assay

The Thermo Pierce Magnetic RNA-Protein pull-down Kit (Thermo Fisher) was used to enriched UCA1-bindign proteins in HaCaT. Magnetic beads were incubated with 50 pM UCA1 probes for 30 min at room temperature. The RNA-protein binding reaction mixture was prepared using 10 μL 10×RNA binding solution, 30 μL 50% glycerol, 30 μL HaCaT cell lysate, 30 μL RNAse, and DNAse free water. The probe-bound beads were incubated with 100 μL of the RNA-protein mixture for 1 h at 4 °C. After removing unbound proteins with 100 μL 1× washing solution provided in the kit, the proteins enriched with UCA1 probes were collected in 50 μL eluent buffer at 37 °C. The biotin-labeled UCA1 probe used in RNA pull-down was synthesized by GenePharma and the sequence was as follows:

5′-GAACATCTCACCAATTTCAAATCGGATCTCCTCGGCTTAGTGGCTGAAGACTGATGCTGCCCGATCGCCTCAGAAGCCCCTTGGACCATCACAGATGCCGAGCTTCGGGTAACTCTTACGGTGGAGGATTCCCAGCCATATGAAGACACCCTAGCTGGACGATCAGTCCTTGTCAAAAGTCTGACCCCTCAAACTCTACAGCCTCAATGGACCAGACCCTACCCGGTCATTTATAGCACACCAACTGCCGTCCATCTGCAGGACCCTCTCCATTGGGTTCACCATTCCAGAATAAAGCCATGCCCATCAGACAGCCAGCTTGATCTCTCCTCTTCCTCCTGGAAGCCACAAGATTAGGCCGAGAGCCGATCAGACAAACAACCTACAACCCTTAAGCTCCTGGCAGCGCCCAGCCAAGGCCATGCTTCCTTGCAACACTCCTTCCAAATGGCCATCCCAGCATGCTTCCAAGCAGGCTTCATCCGTTCCTCTGGACCCTCATCTCTTAAGACCTGCCGCCTATAAAAAGGATTATATCTTGAGACCCTATCCTCTAAAATTTTTTCCACACCCAA AACA-3′

### Statistical analysis

Statistical analysis was conducted using Excel, GraphPad Prism (version 8.0), and R software. Data were presented as mean ± standard deviation (SD) of at least three independent experiments or three replicates. Two-sided student’s t-test or Mann-Whitney test was used to compare two groups, and analysis of variance (ANOVA) was used to compare multiple groups. The FDR method was used to correct *P* values in omics data. *P* values less than 0.05 were considered statistically significant and shown as asterisk (*P < 0.05, **P < 0.01, ***P < 0.001, ****P < 0.0001, ns: not significant. Some *P* values of Pearson correlation analysis were too small to calculate in R and were shown as 0). Graphs were plotted using R and GraphPad Prism, and Photoshop. Krita, and Adobe Illustrator were used for typesetting.

## Supplementary information

Supplementary materials

Raw images of western blots

Reproducibility checklist

## Data Availability

The GEO datasets used in this study are available at the www.ncbi.nlm.nih.gov/geo. Data generated or analyzed during this study and R scripts are included in this manuscript. Supplementary files are available from the corresponding author upon reasonable request.
