# Supplementary material for: LncRNA UCA1 promotes keratinocyte-driven inflammation via suppressing METTL14 and activating the HIF-1α/NF-κB axis in psoriasis
Source: Cell Death Dis. 2023 Apr 20;14(4):279. doi: 10.1038/s41419-023-05790-4 (PMC10115875; doi:10.1038/s41419-023-05790-4)
Supplement: Supplementary file 1 — Supplementary materials [file 41419_2023_5790_MOESM1_ESM.docx]

**Supplementary**

Contents: 1 table and 13 figures.

**Table. S1** Information of GEO datasets

| GEO accession | Species | Sample size | Treatments | Platform | Type | Date | PMID |
| --- | --- | --- | --- | --- | --- | --- | --- |
| GSE117468 | human | 565 | Brodalumab  Ustekinumab | Microarray | transcriptome | 2018 | 31883845 |
| GSE13355 | human | 180 | none | Microarray | transcriptome | 2008 | 19169254 |
| GSE14905 | human | 82 | none | Microarray | transcriptome | 2009 | 18648529 |
| GSE30999 | human | 170 | none | Microarray | transcriptome | 2011 | 22763790 |
| GSE41664 | human | 157 | Etanercept | Microarray | transcriptome | 2012 | 23308107 |
| GSE52471 | human | 31 | none | Microarray | transcriptome | 2013 | 23771123 |
| GSE53552 | human | 99 | Brodalumab | Microarray | transcriptome | 2013 | 24646743 |
| GSE54456 | human | 174 | none | Sequencing | transcriptome | 2014 | 24441097 |
| GSE155702 | human | 24 | none | MeRIP | m6A | 2020 | 33251217 |


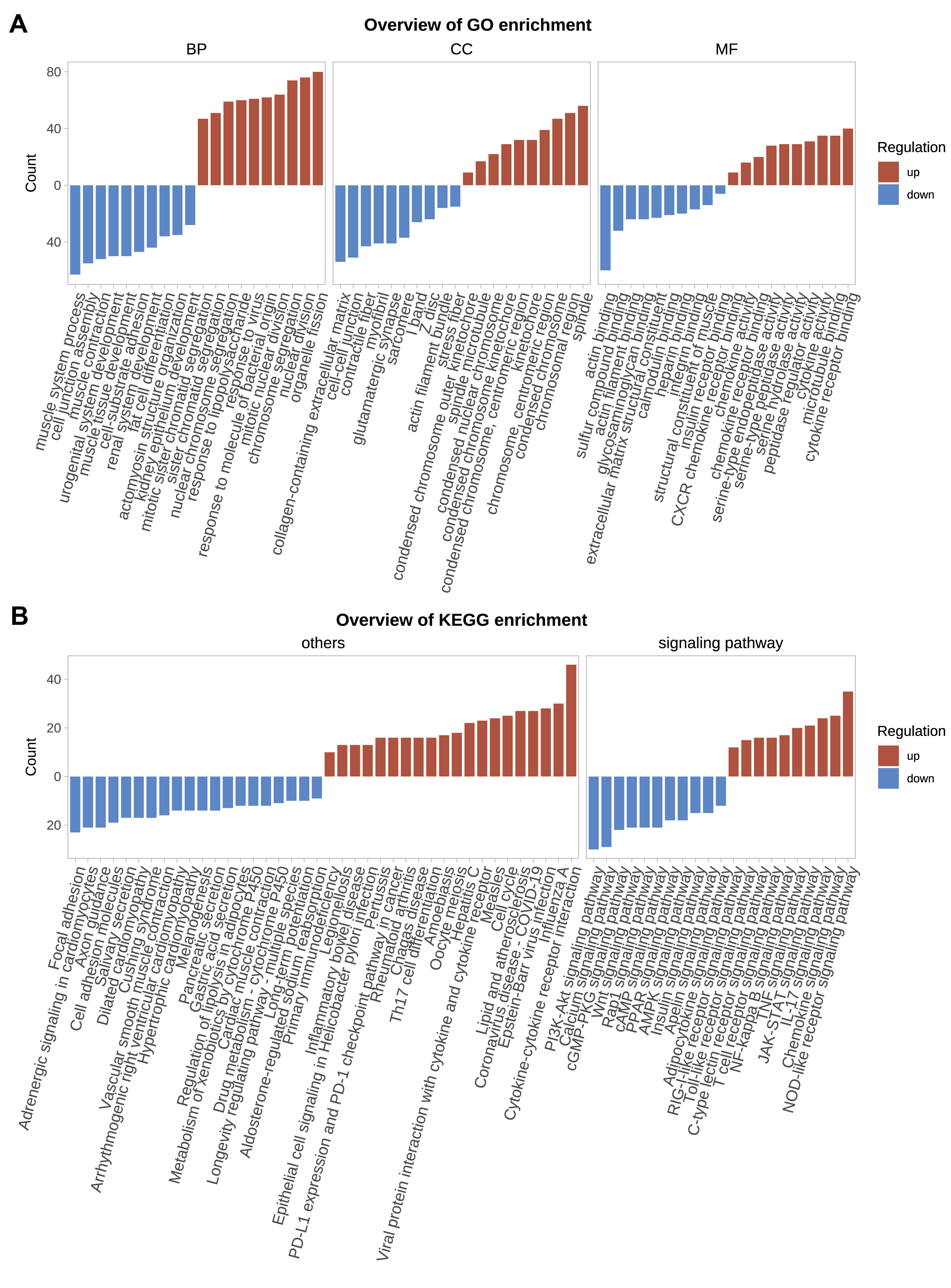


**Figure. S1 Go & KEGG enrichment of merged transcriptome data from GEO datasets.**


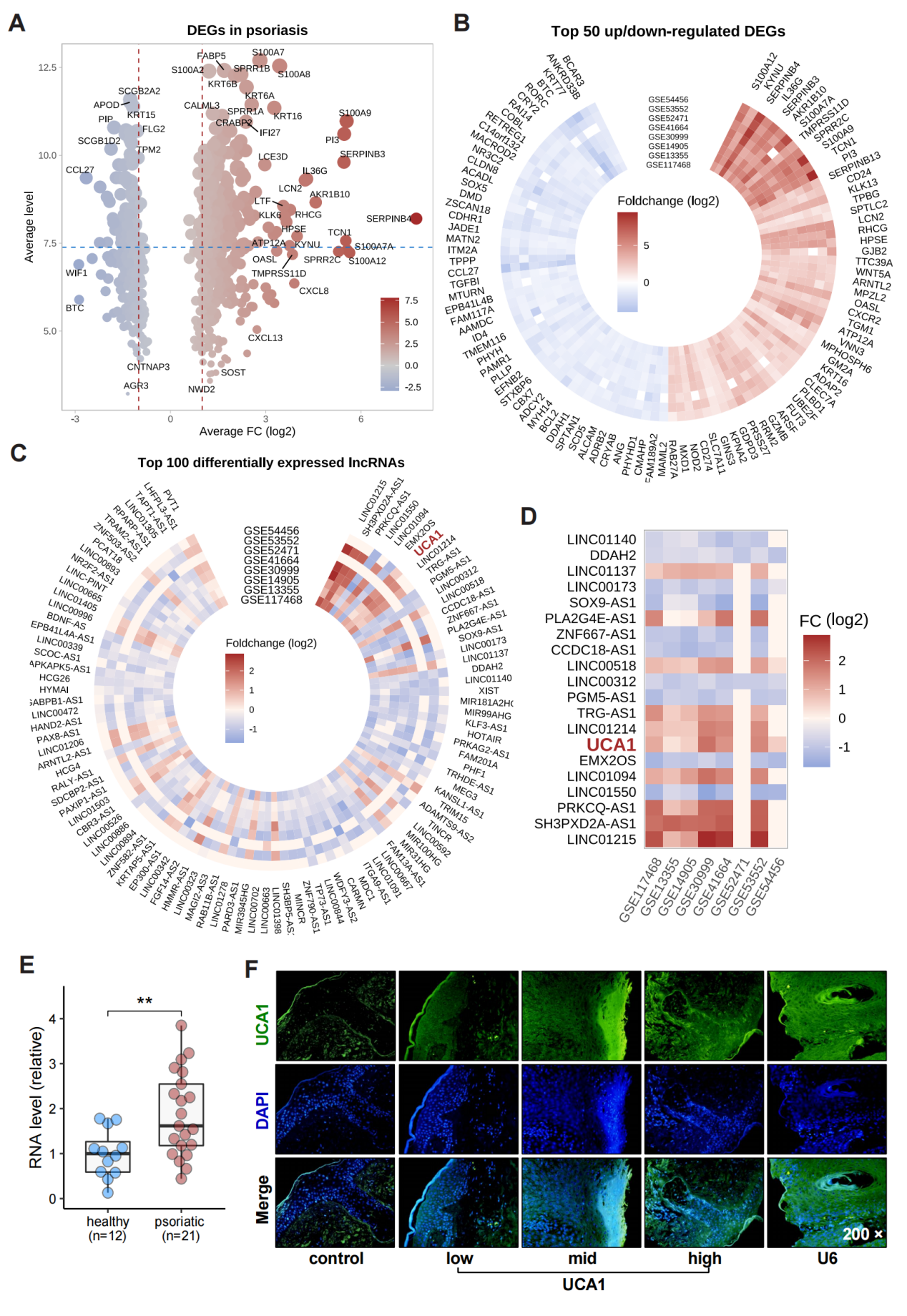


**Figure. S2 DEGs in 8 GEO datasets.** A. Average expression levels and log_2_ foldchange (psoriatic lesions *vs* normal skin) of genes. B. Top ranked DEGs. C-D. Top ranked lncRNAs. E. UCA1 levels in skin samples were detected by qPCR. F. RNA levels in psoriatic lesions were detected by FISH (magnification: 200×).


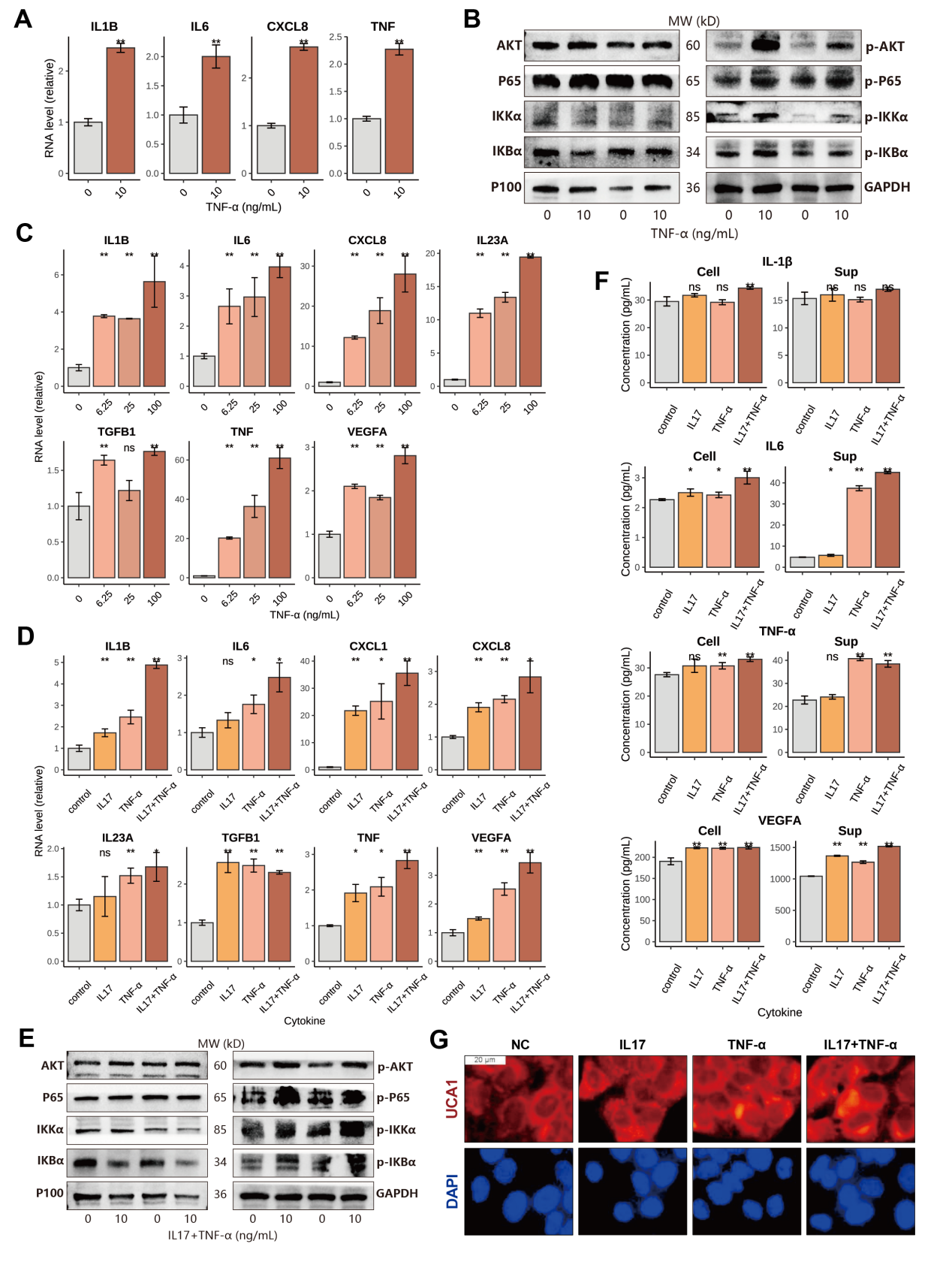


**Figure. S3 Cytokines IL17 and TNF-α were used to induce inflammation in HEK and HaCaT.** A. RNA levels of genes in HEK treated with TNF-α (10 ng/mL). B. Protein levels of signaling pathways in HEK treated with TNF-α. C-D. RNA levels of genes in HaCaT treated with TNF-α (6.25-100 ng/mL) or IL17+TNF-α (10 ng/mL). E. Protein levels of signaling pathways in HaCaT. F. Cytokine levels in HaCaT supernatant. G. UCA1 levels were detected using FISH in IL17+TNF-α (10 ng/mL) treated HaCaT (white scale bar: 20 μm).


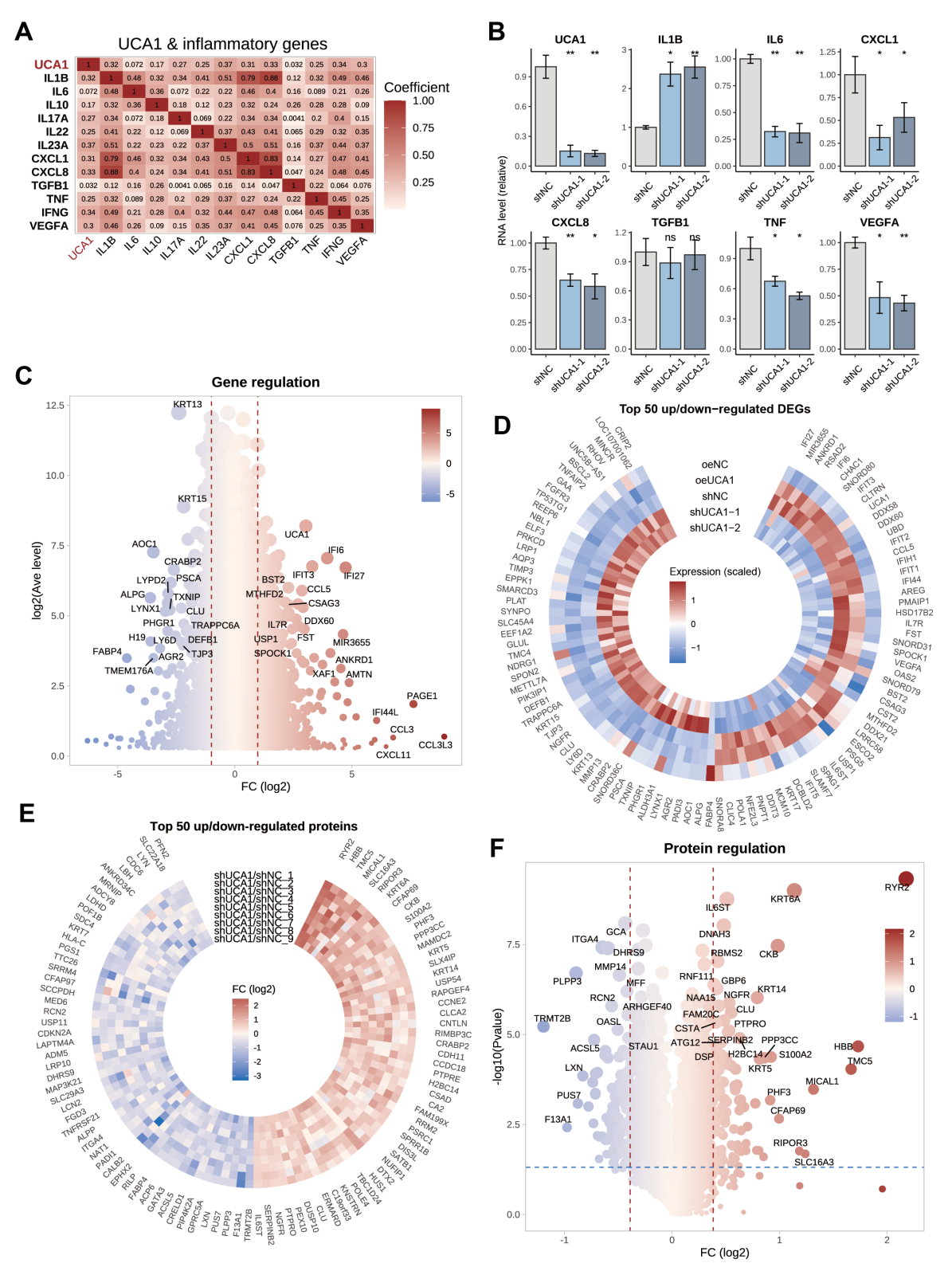


**Figure. S4 HaCaT transcriptome and proteomics data.** A. The correlation (Pearson correlation coefficient) between UCA1 and inflammatory genes in GEO datasets. B. The RNA levels of inflammatory gene in UCA1-knockdown HaCaT. C. Gene regulation in transcriptome data of HaCaT (the log_2_-transferred foldchange of DEGs in UCA1 over-expressed or knockdown cells was weighted: log_2_FC_weighted_ = log_2_FC_oe_ - log_2_FC_sh_). D. The expression of top ranked genes in transcriptome data. E. The ratio (shUCA *vs* shNC) of top ranked proteins in proteomic data. F. Protein regulation in proteomic data of UCA1-knockdown HaCaT. (UCA1 over-expressed: oeUCA1; UCA1 knockdown: two different short hairpin RNAs shUCA1-1 & shUCA1-2)


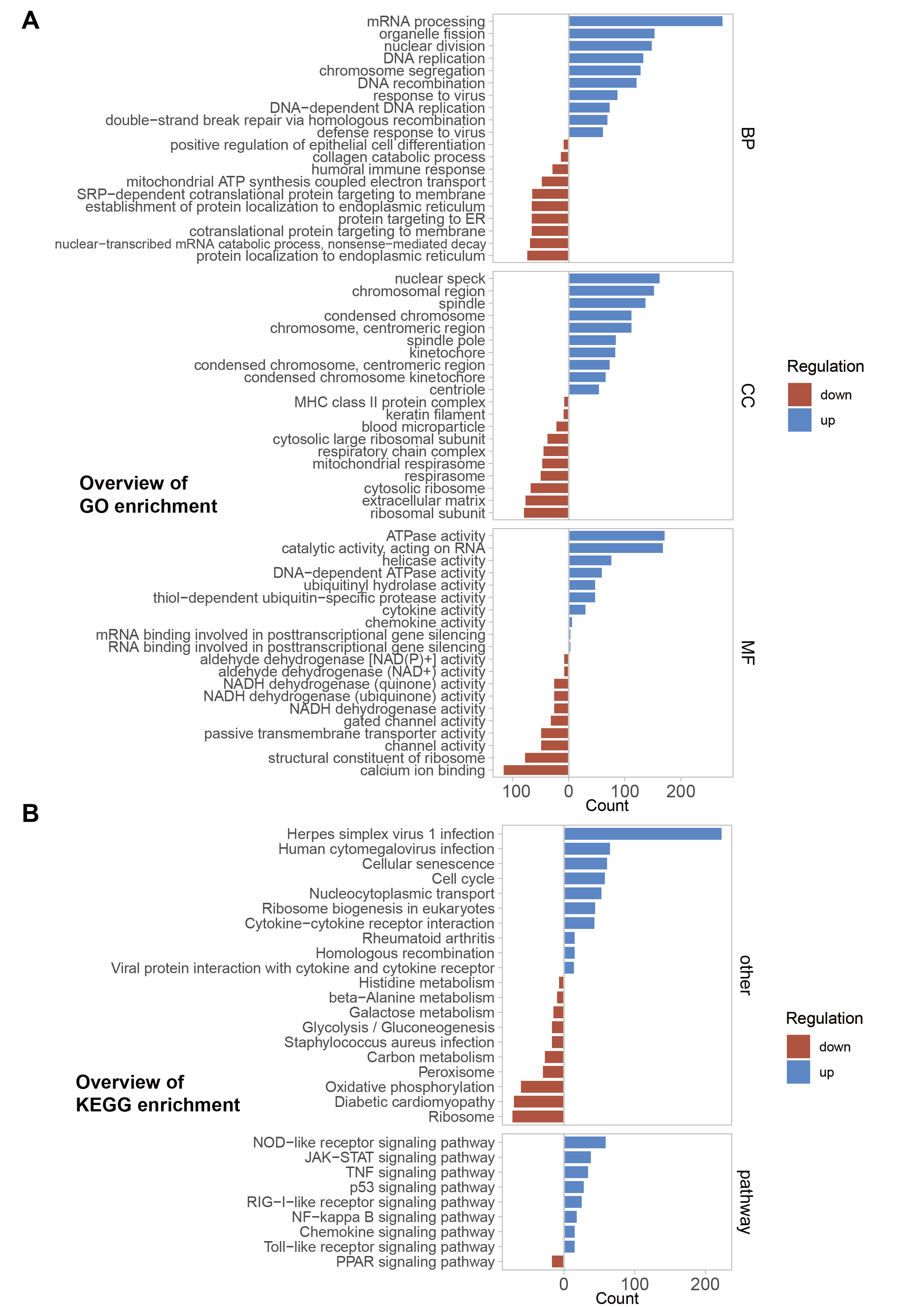


**Figure. S5 Go & KEGG enrichment of UCA1-overexpressed or knockdown HaCaT transcriptome.** (The log_2_-transferred foldchange of DEGs in UCA1 over-expressed or knockdown cells was weighted: log_2_FC_weighted_ = log_2_FC_oe_ - log_2_FC_sh_)


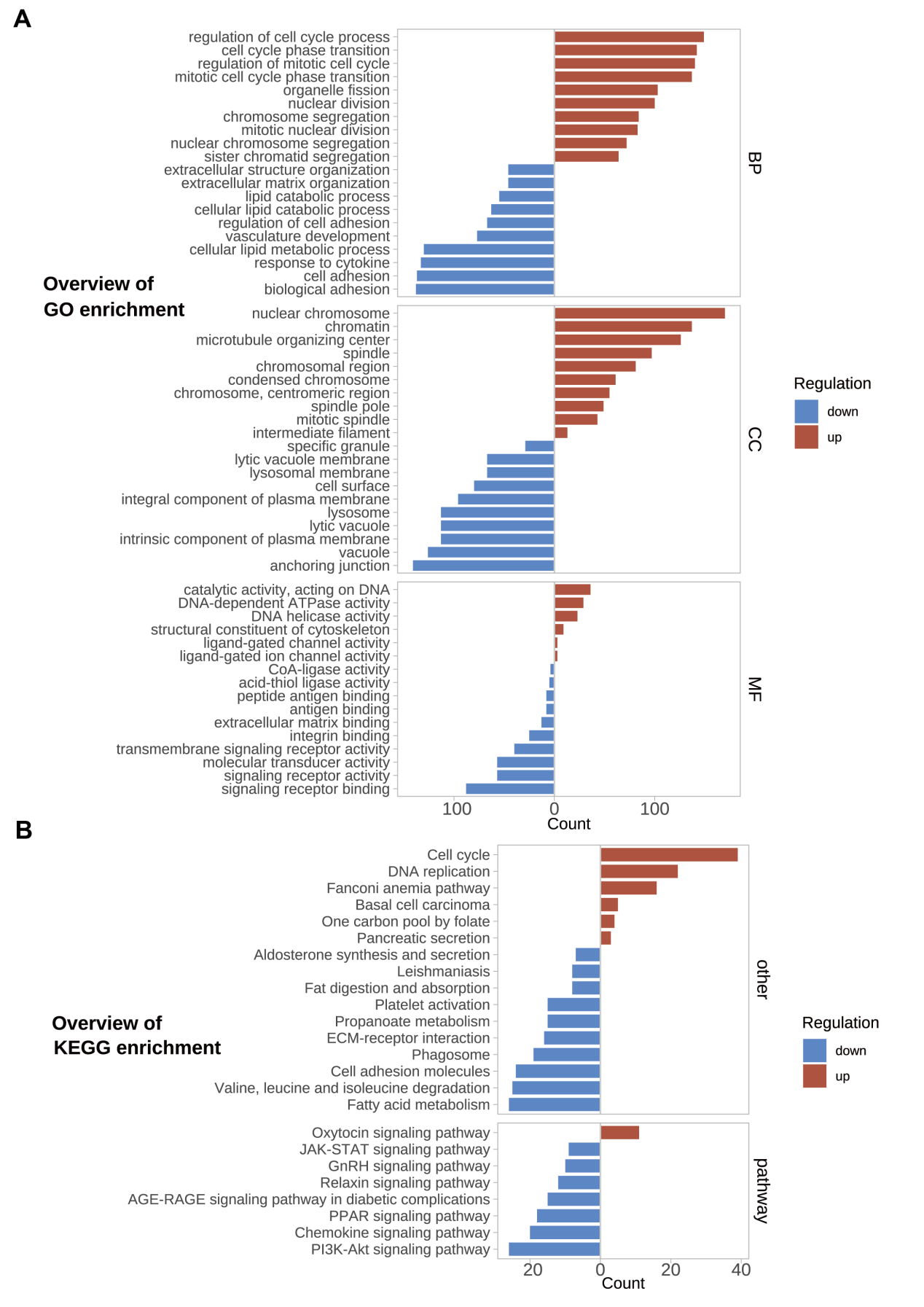


**Figure. S6 Go & KEGG enrichment of UCA1-knockdown HaCaT proteomic data.**


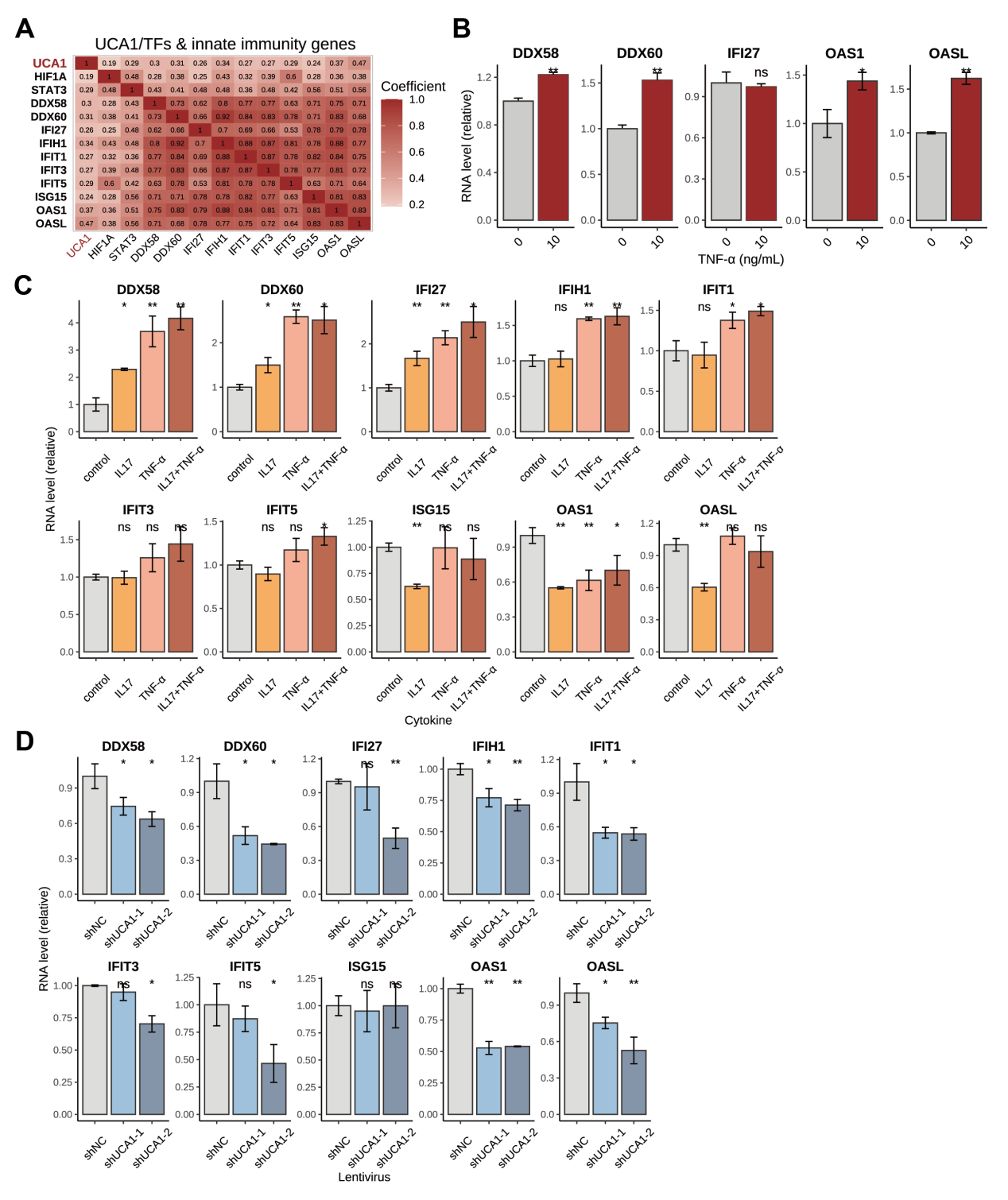


**Figure. S7 The relation between UCA1 and inflammation and innate immunity.** A. Pearson correlation between UCA1 and innate immunity-related gene expression in GEO data. B. RNA levels of innate immunity-related genes in HEK. C. RNA levels of innate immunity-related genes in HaCaT treated with IL17+TNF-α (10 ng/mL). D. RNA levels of innate immunity-related genes in UCA1-knockdown HaCaT. (UCA1 knockdown: two different short hairpin RNAs shUCA1-1 & shUCA1-2)


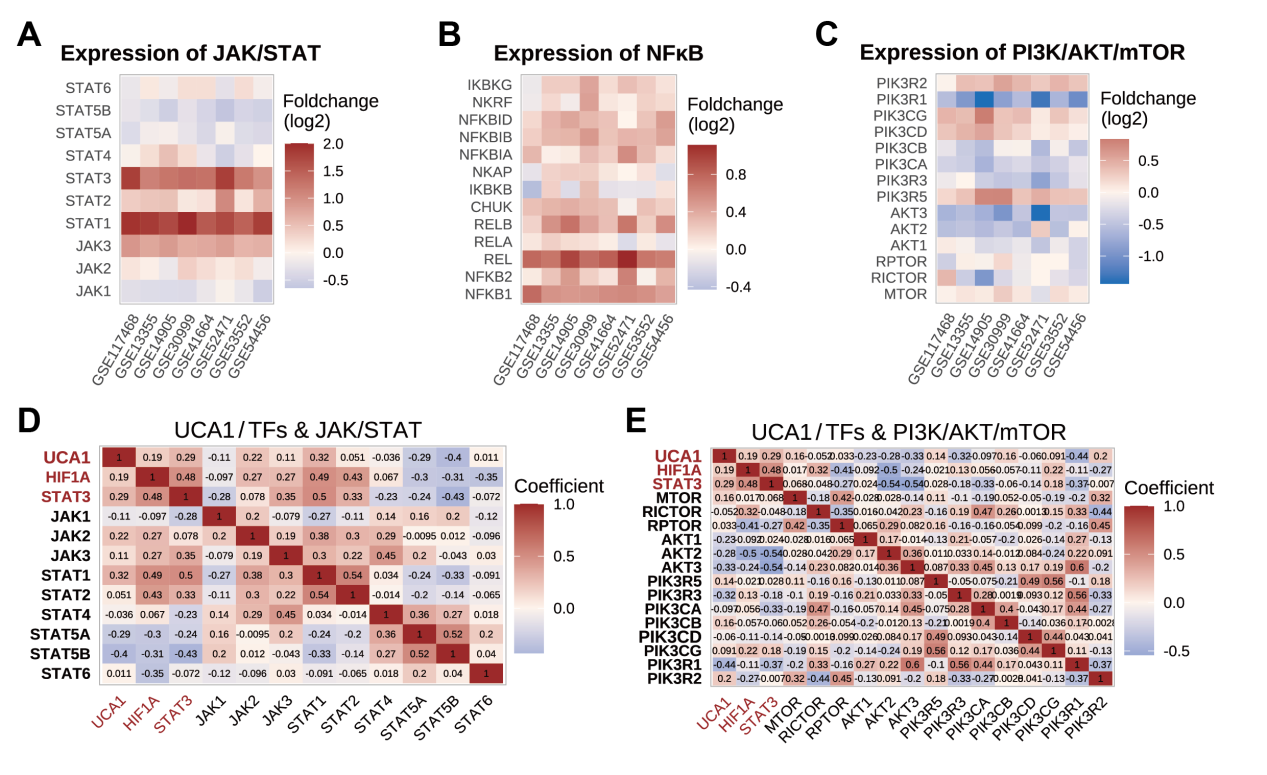


**Figure. S8** The relation between UCA1 and inflammatory signaling pathways in GEO data. A-C. Expression of pathway genes. D-E. Pearson correlation between UCA1 and pathway genes.


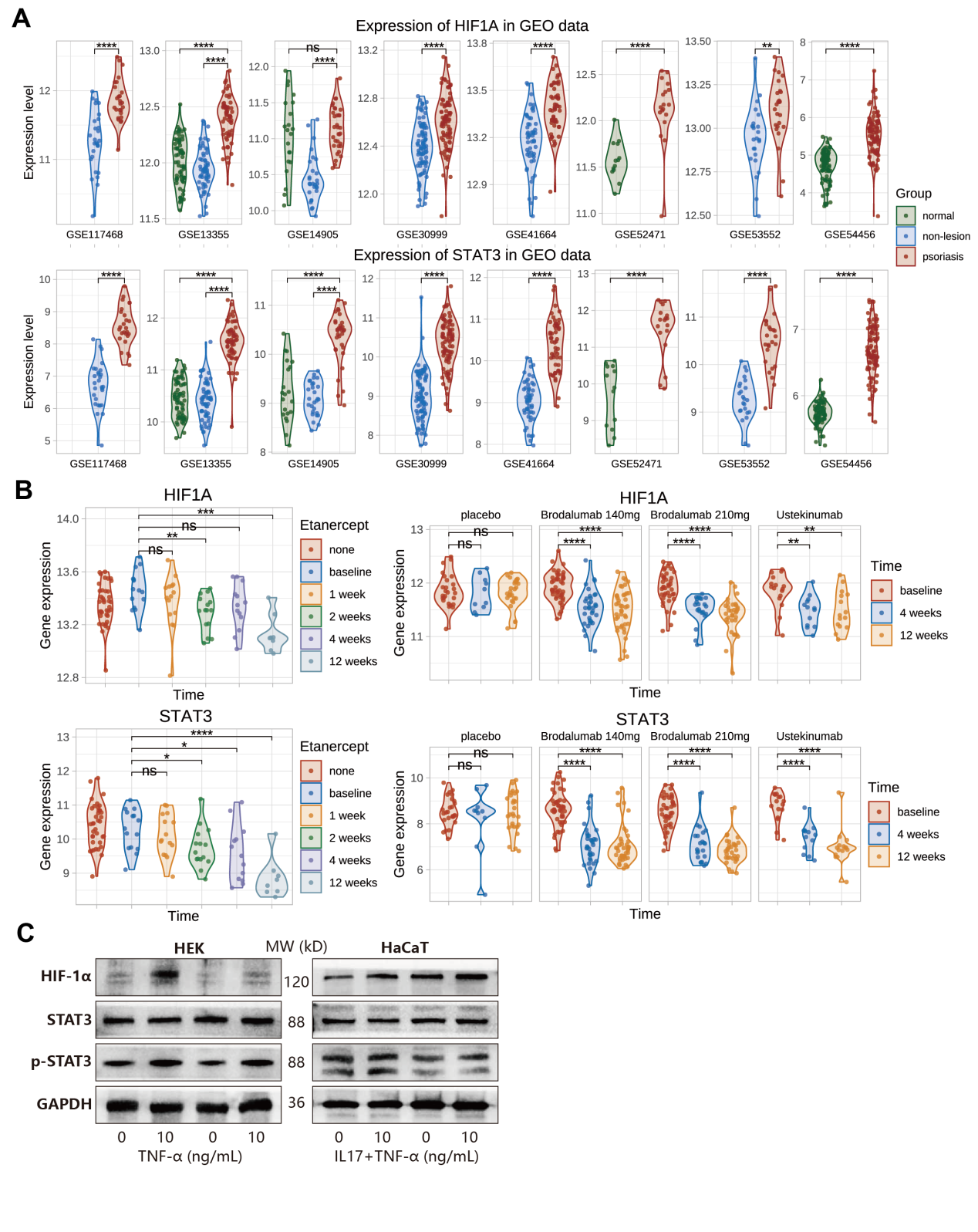


**Figure. S9** The relation between HIF1A, STAT3 and psoriasis. A. HIF1A and STAT3 expression in GEO datasets. B. HIF1A and STAT3 expression under biologics treatments (GSE41664 and GSE117468). C. Protein levels of HIF-1α and STAT3 in HaCaT treated with IL17+TNF-α (10 ng/mL).


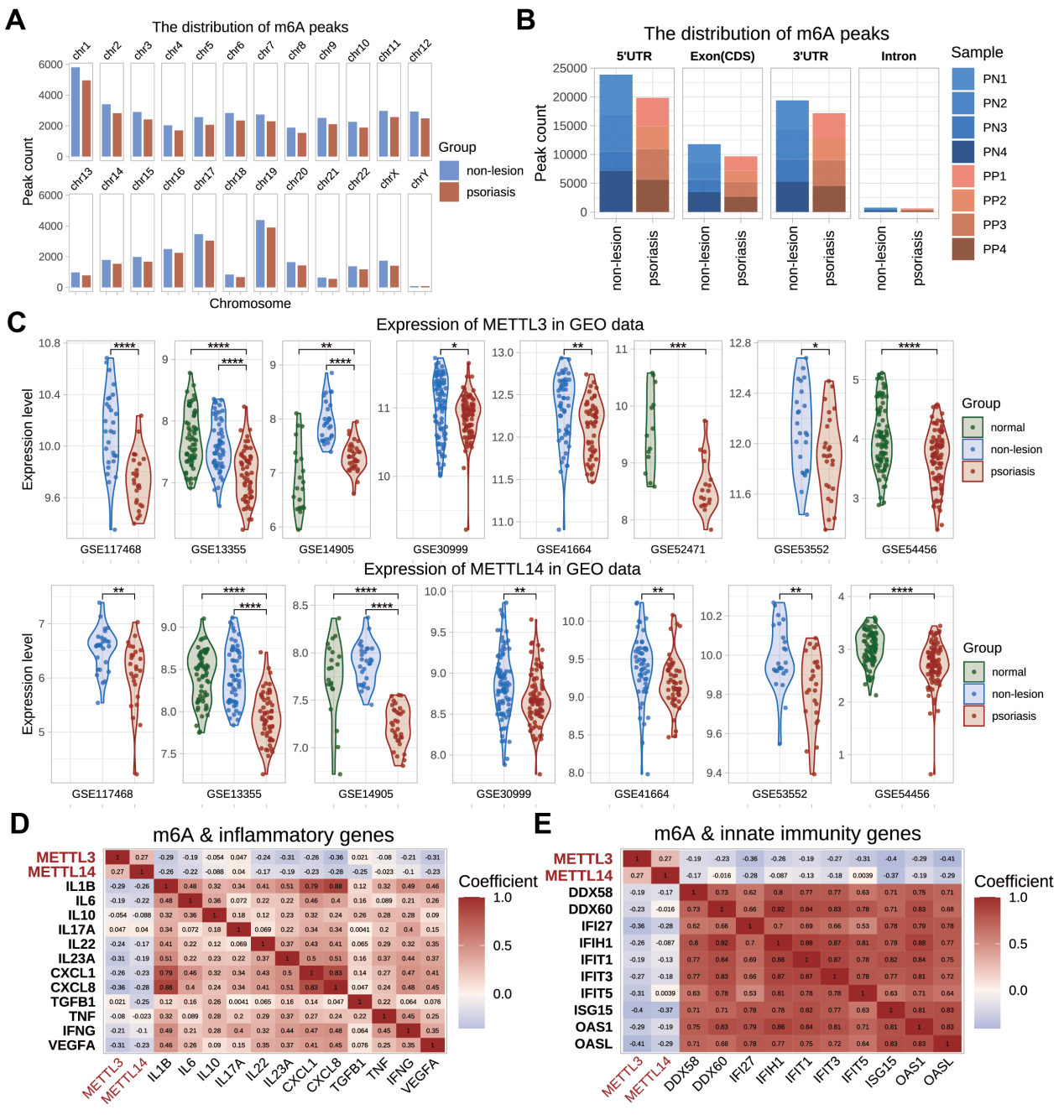


**Figure. S10 The relation between m^6^A-related genes and psoriasis.** A-B. The amount and distribution of m^6^A modification peaks in psoriatic and normal skin. C. METTL3 and METTL14 expression in GEO datasets. D-E. Pearson correlation between METTL3, METTL14, inflammatory genes, and innate immunity-related genes.


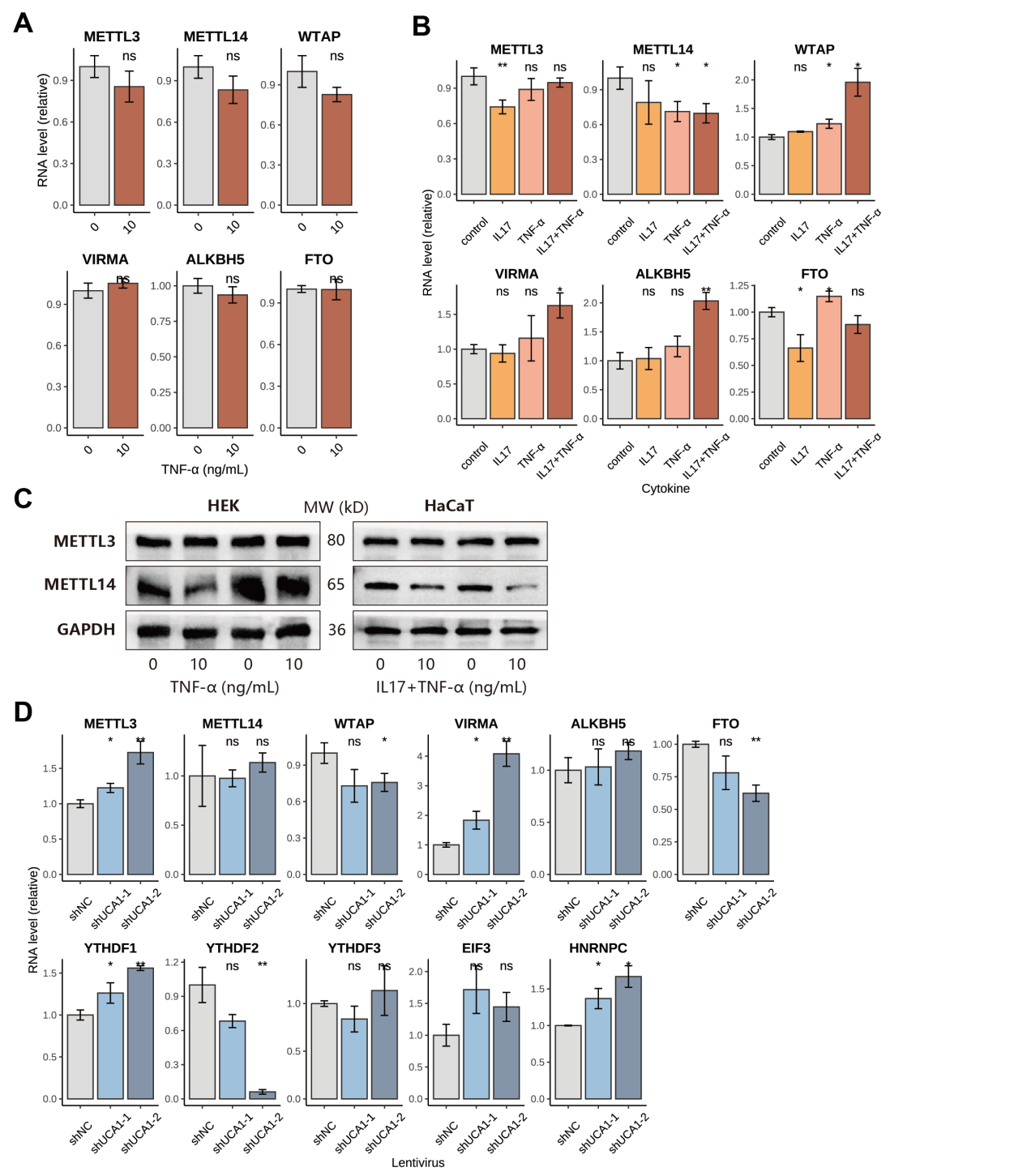


**Figure. S11 METTL3 and METTL14 expression in inflammatory or UCA1-knockdown keratinocytes.** A. RNA levels of genes in HEK treated with TNF-α. B-C. RNA and protein levels of genes in HaCaT treated with IL17+TNF-α (10 ng/mL). C. RNA levels of genes in UCA1-knockdown HaCaT.


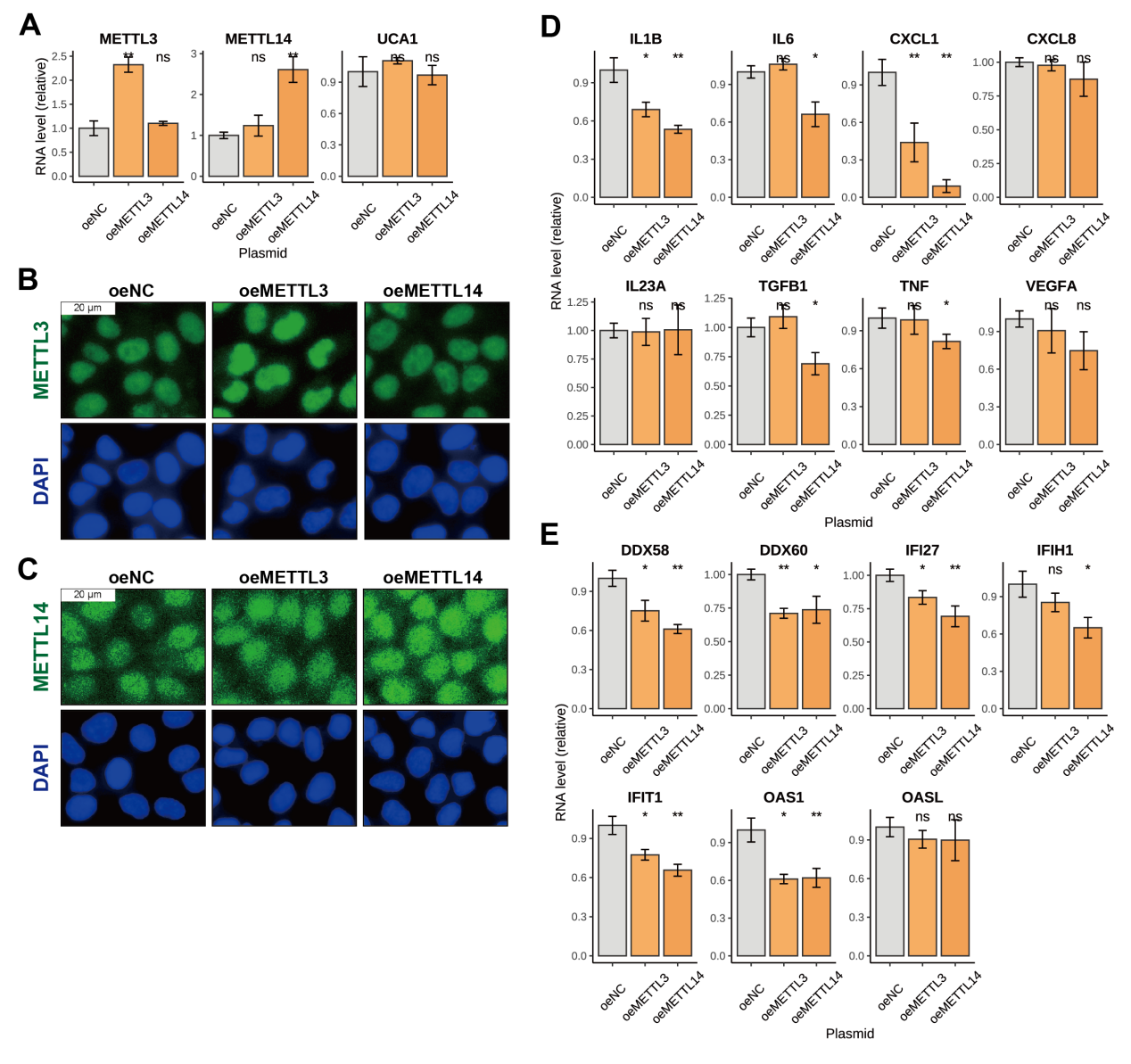


**Figure. S12 The effects of METTL3 or METTL14 over-expression in HaCaT.** A. RNA levels of genes. B-C. Protein levels and distribution detected by IF. D-E. RNA levels of inflammatory genes and innate immunity-related genes.


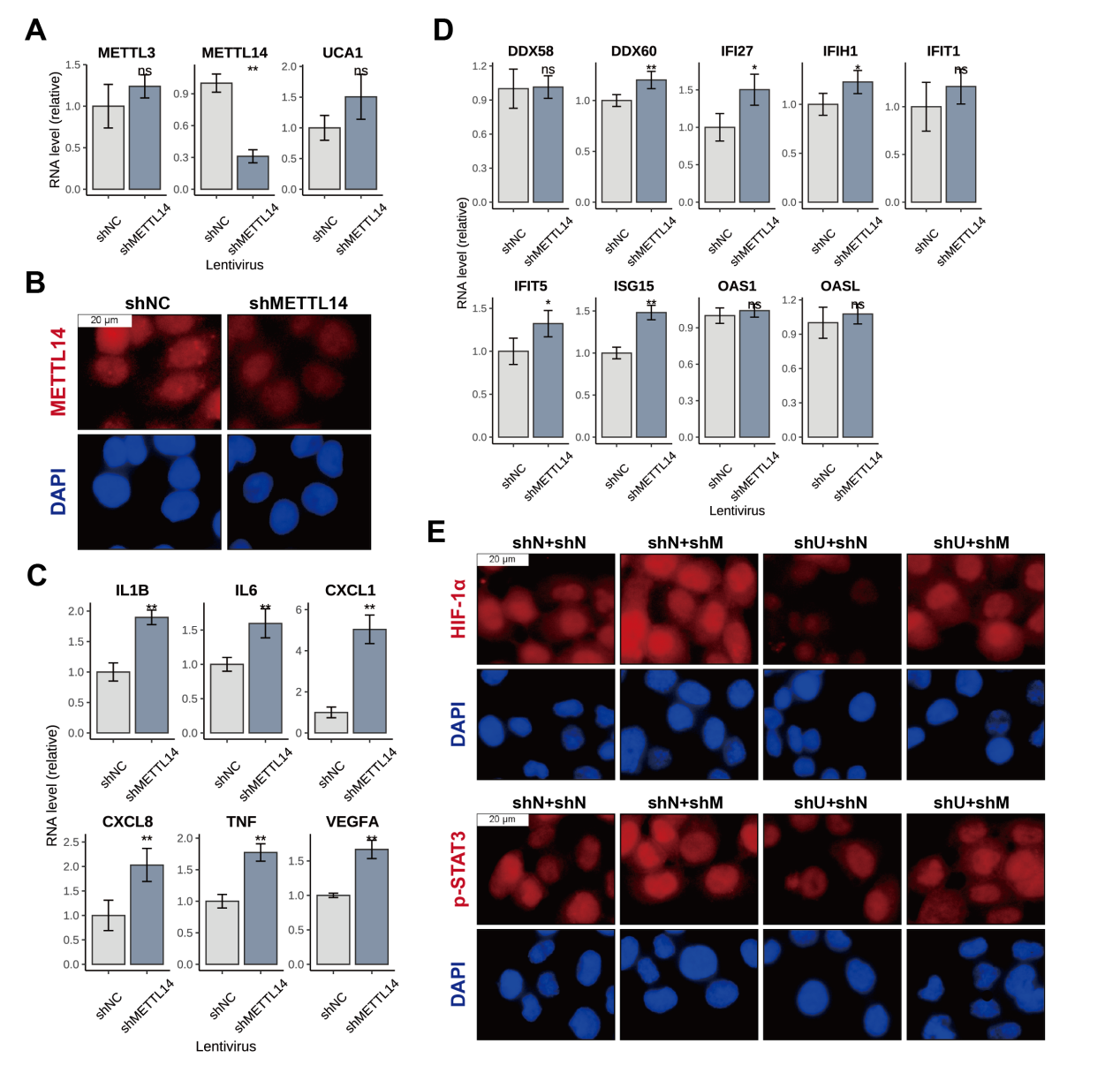


**Figure. S13 The effects of METTL14 knockdown in HaCaT.** A. RNA levels of genes. B. Protein levels and distribution detected by IF. C-D. RNA levels of genes. E. Protein levels and distribution in UCA1 and METTL14 co-transfected HaCaT (shN: knockdown control, shU: UCA1 knockdown, shM: METTL14 knockdown).
