# Supplementary figures and images for: LncRNA UCA1 promotes keratinocyte-driven inflammation via suppressing METTL14 and activating the HIF-1α/NF-κB axis in psoriasis
Source: Cell Death Dis. 2023 Apr 20;14(4):279. doi: 10.1038/s41419-023-05790-4 (PMC10115875; doi:10.1038/s41419-023-05790-4)

Fig. 3F


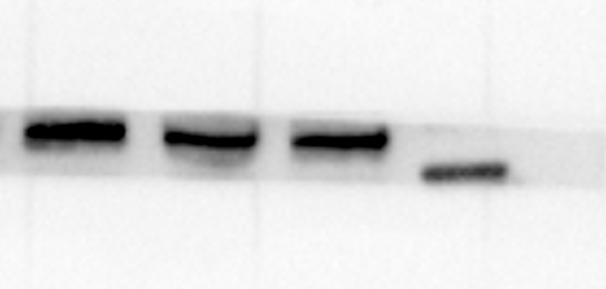


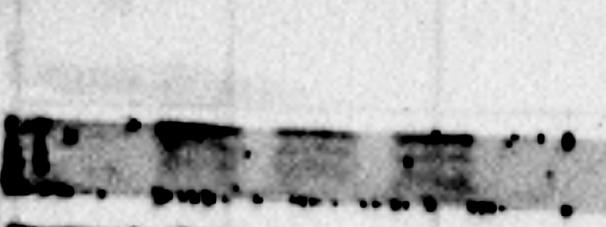


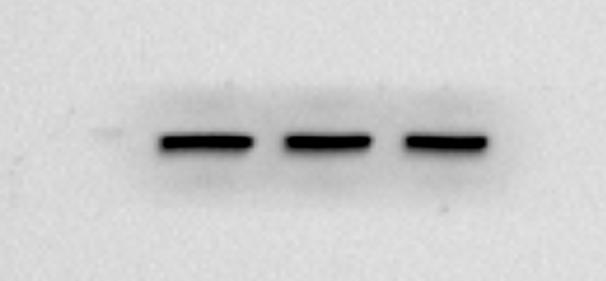


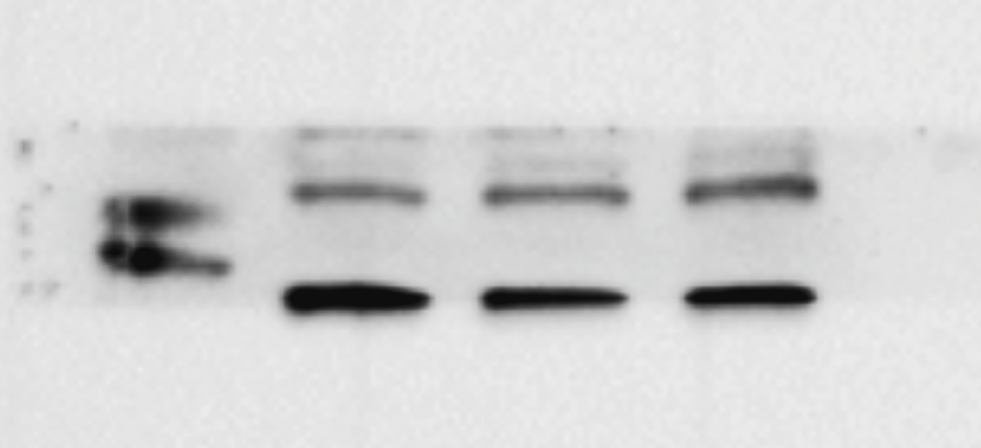


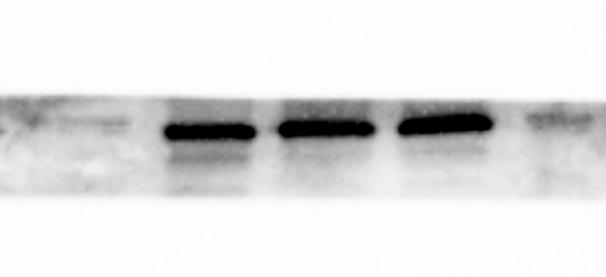


Fig. 4C


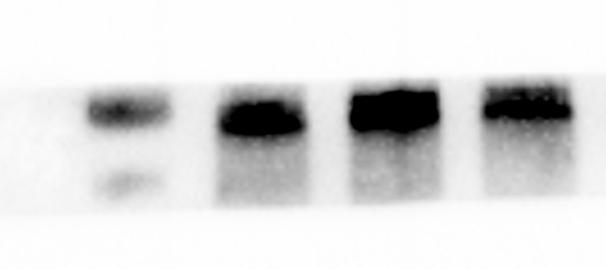

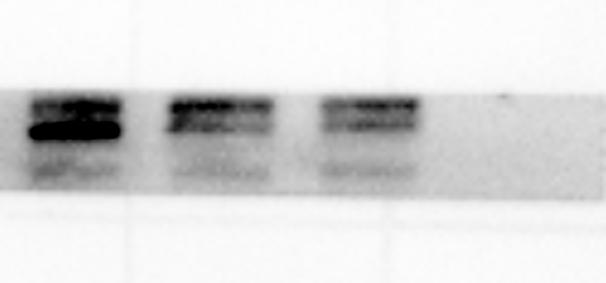


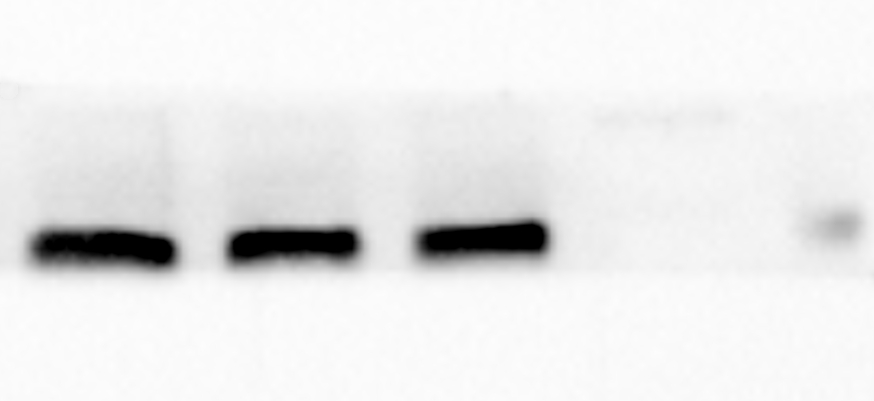

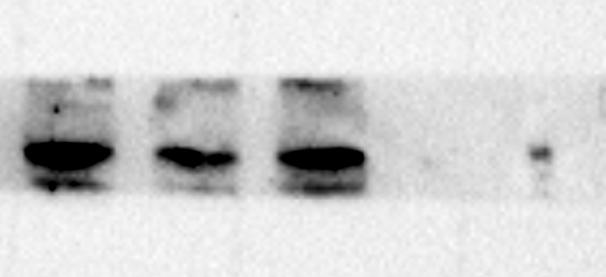


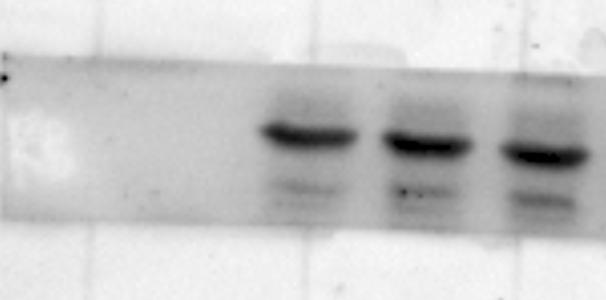

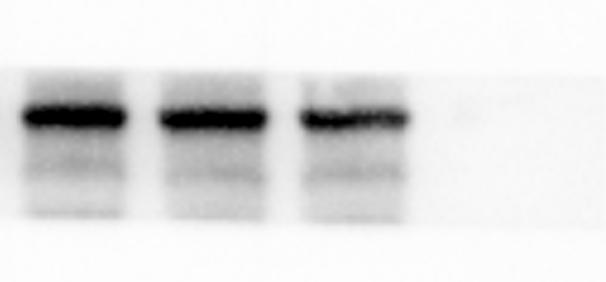


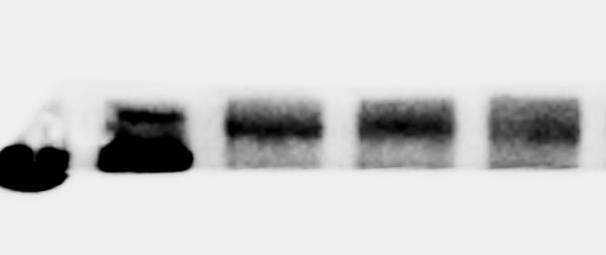

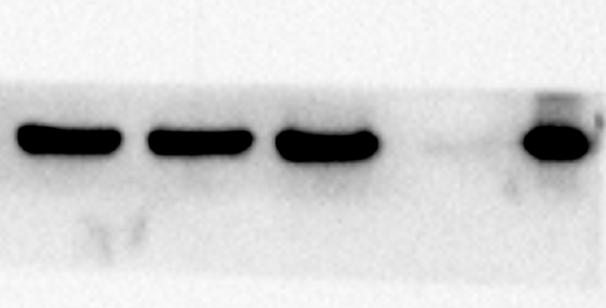


Fig. 4H


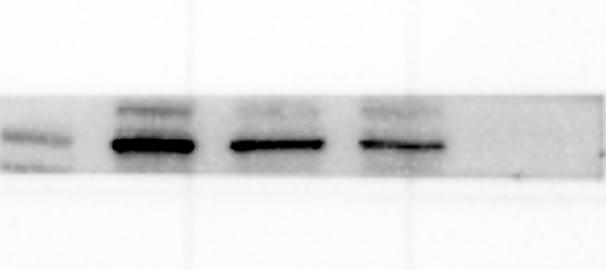


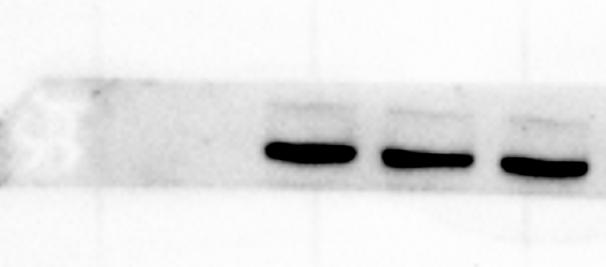


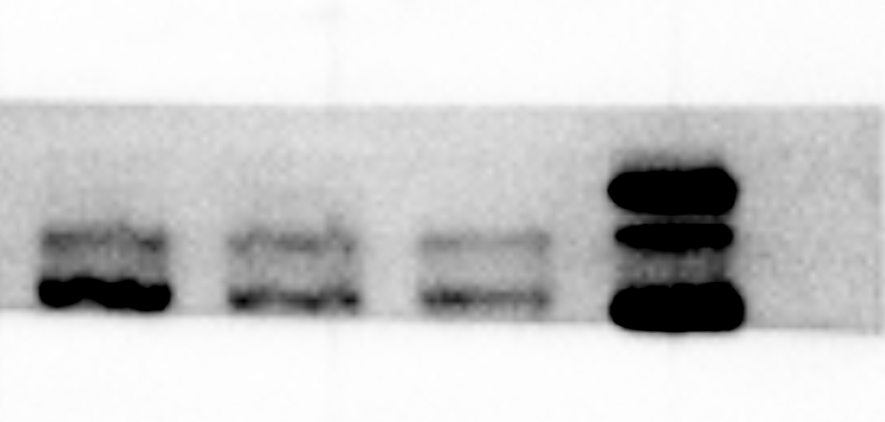


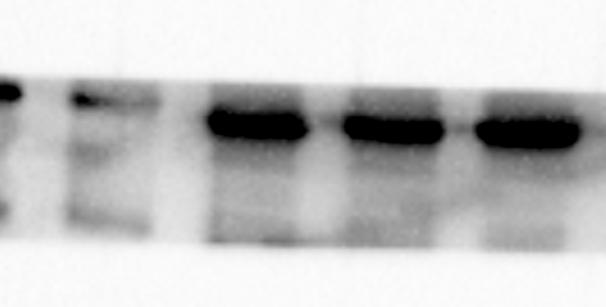


Fig. 5F


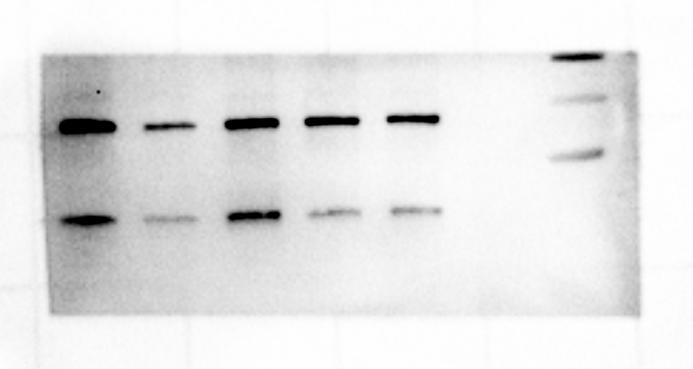


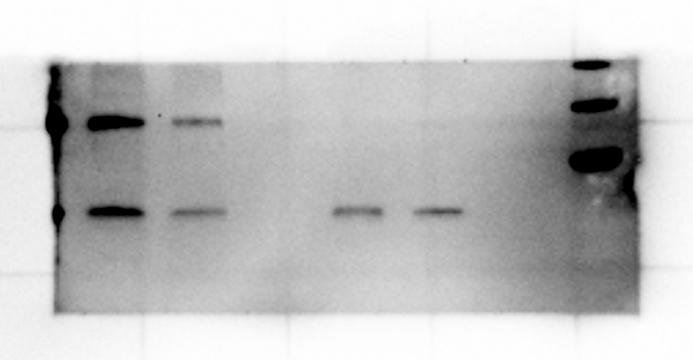


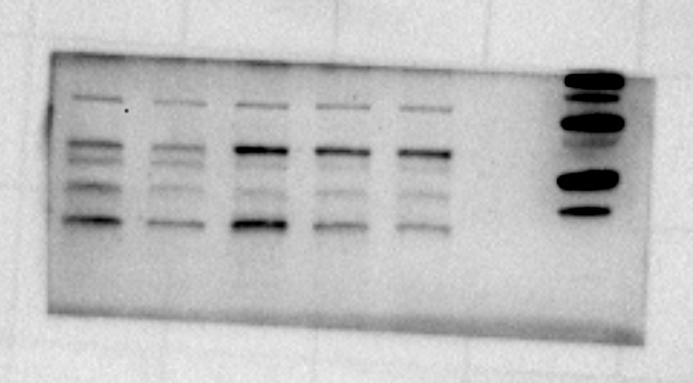


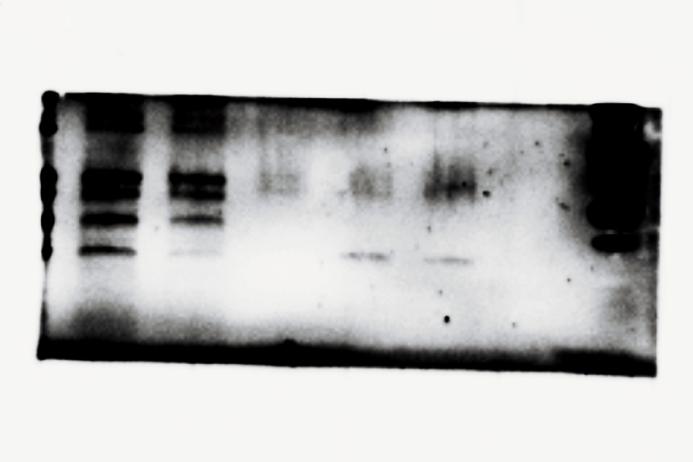


Fig. 6B


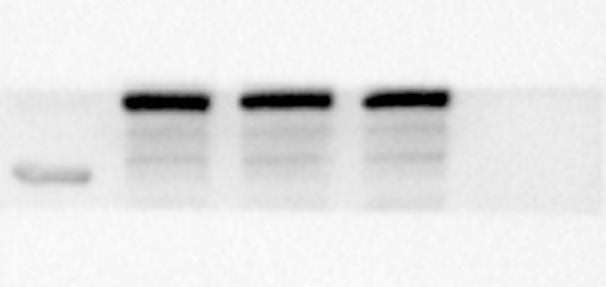

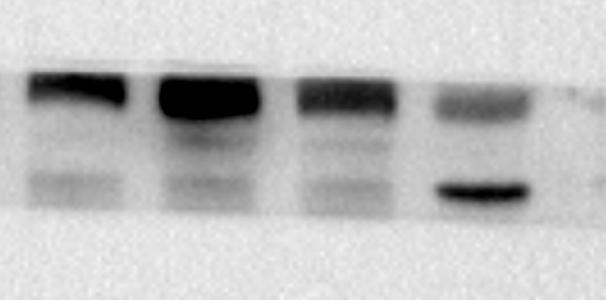


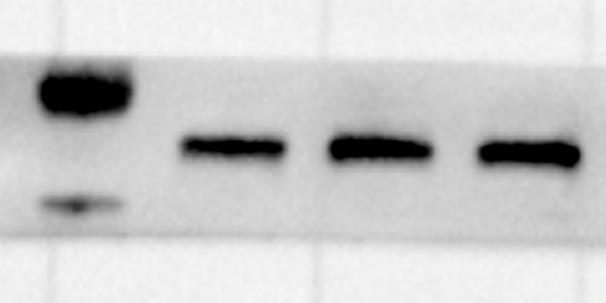

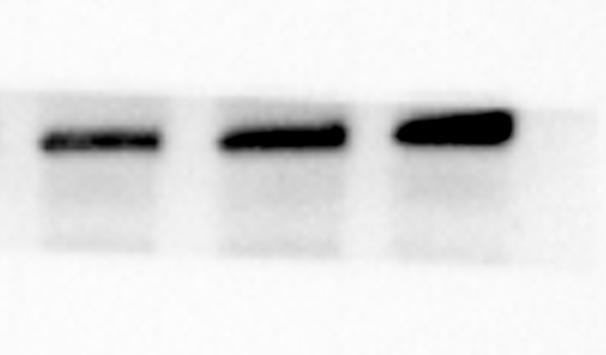


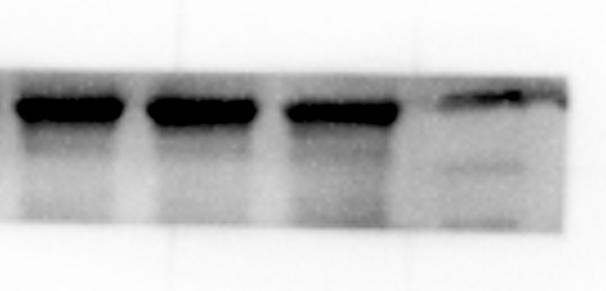

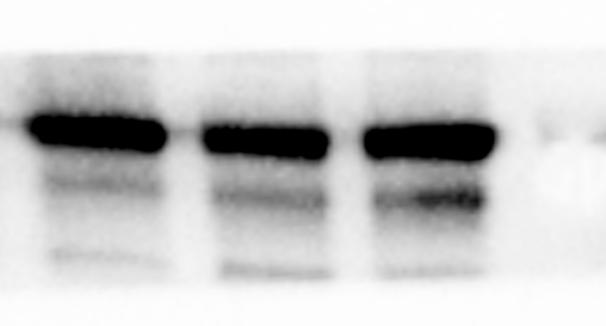


Fig. 6D


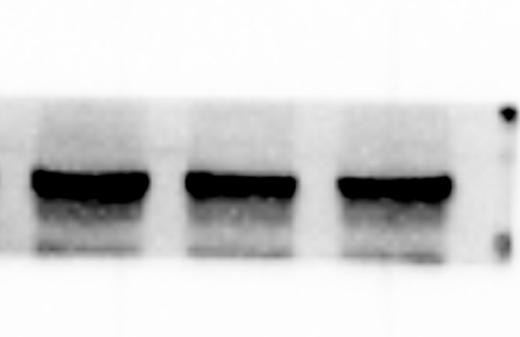


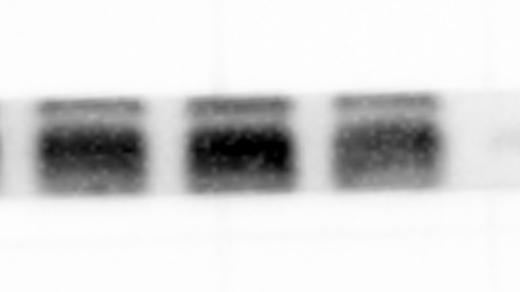


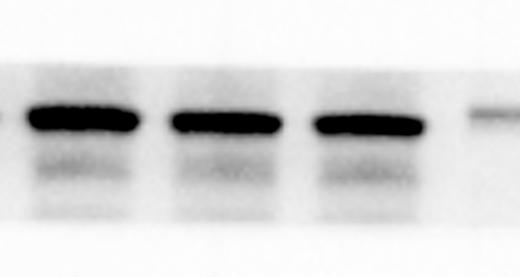


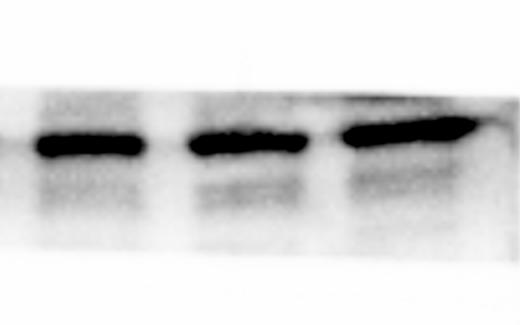


Fig. 6E


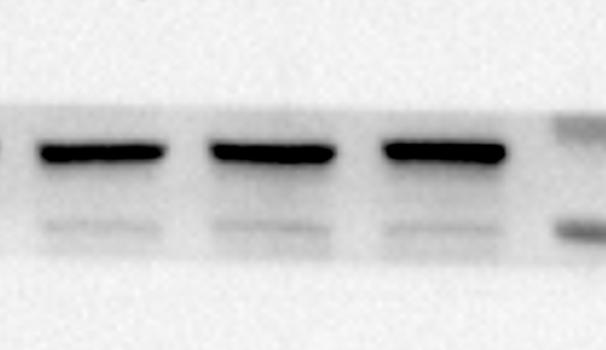

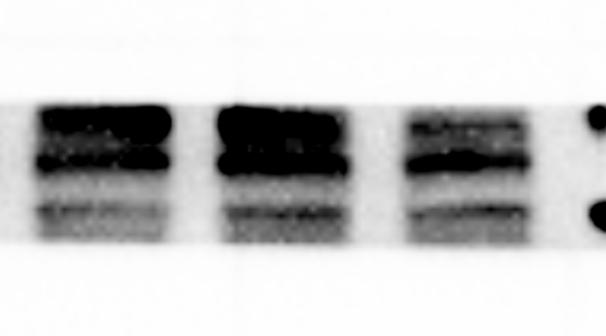


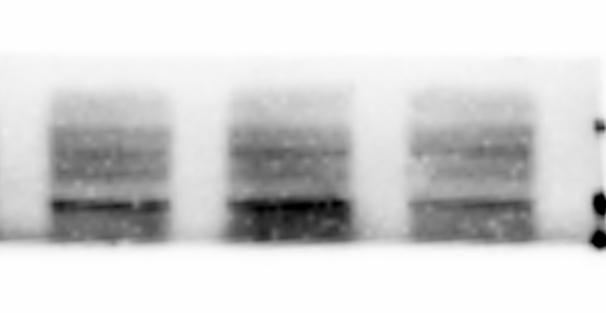

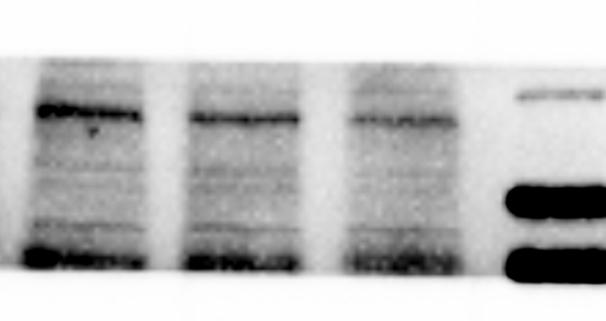


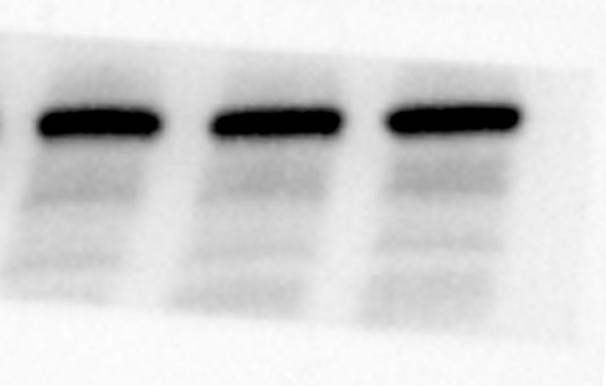

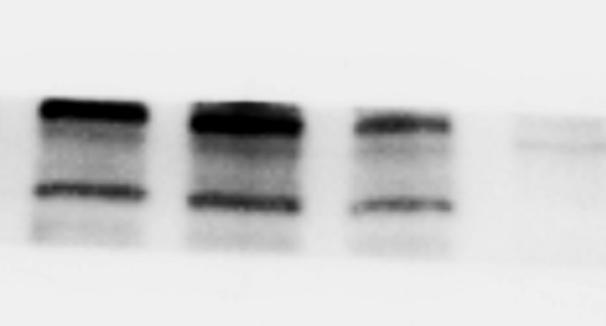


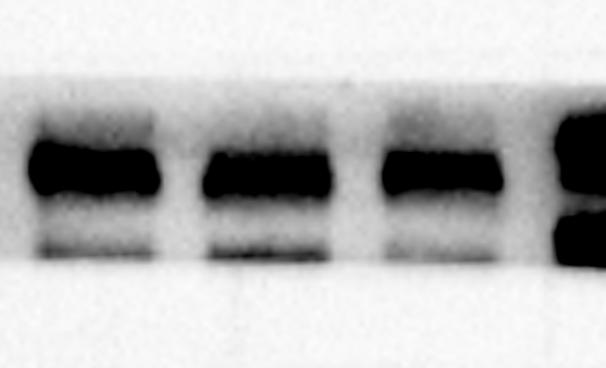

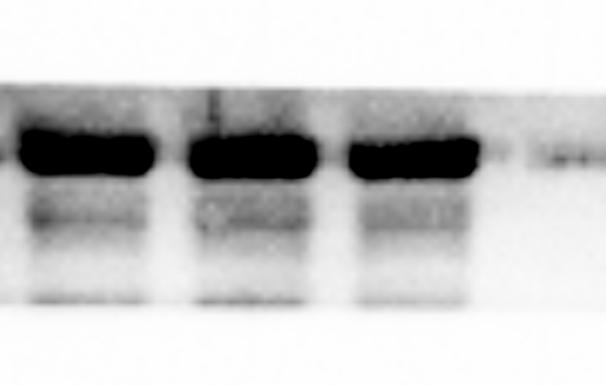


Fig. 6F


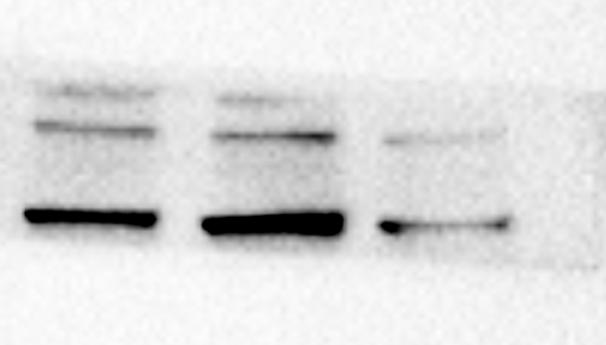


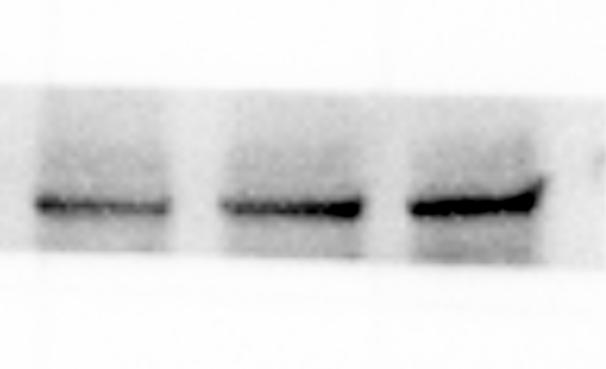


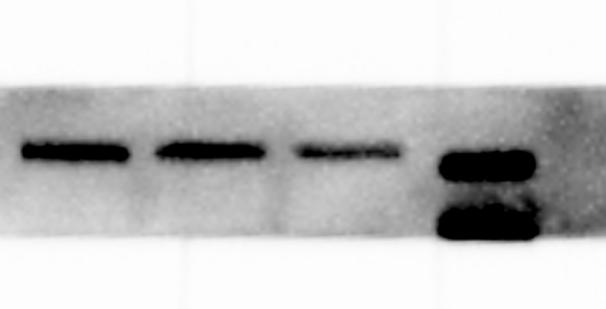


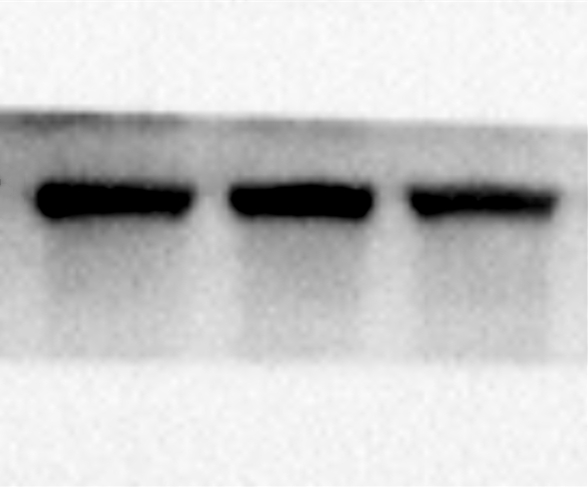


Fig. 7A


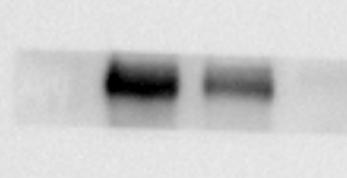


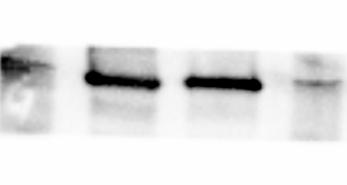


Fig. 7C


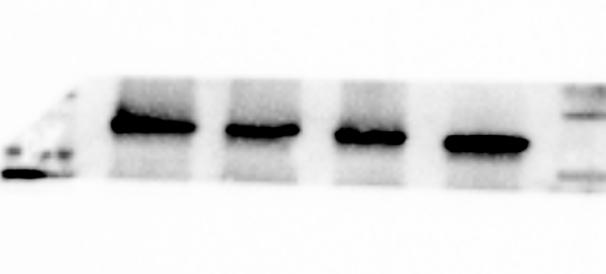


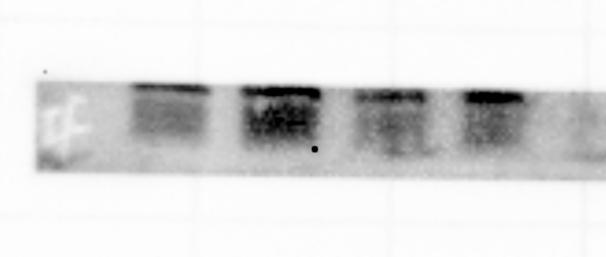


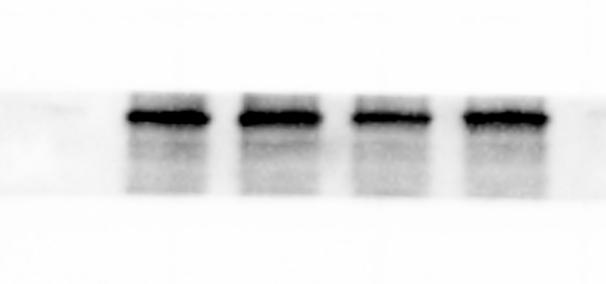


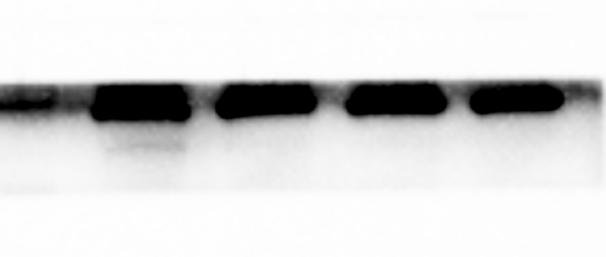


Fig. 7D


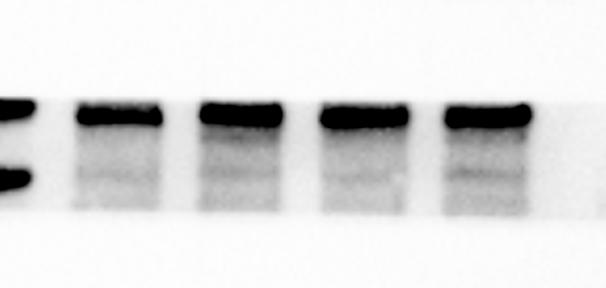

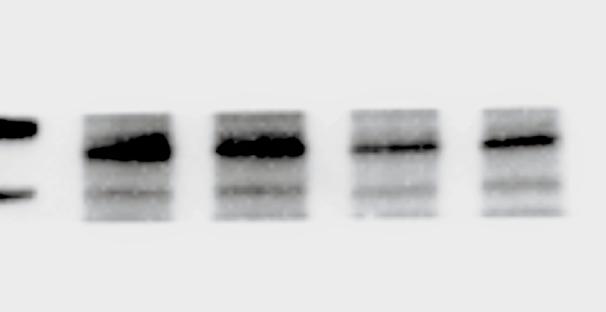


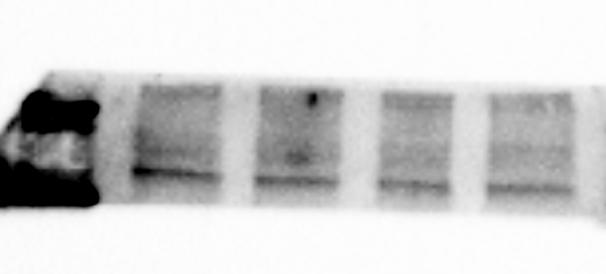

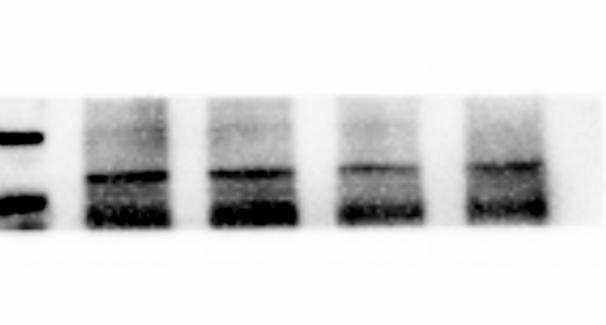


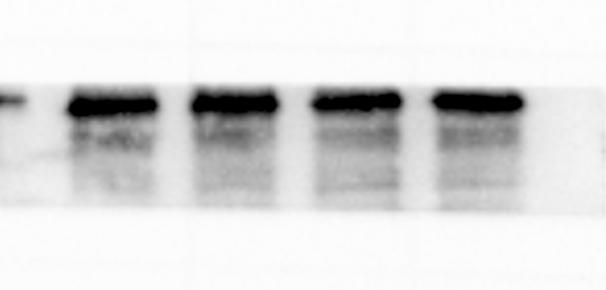

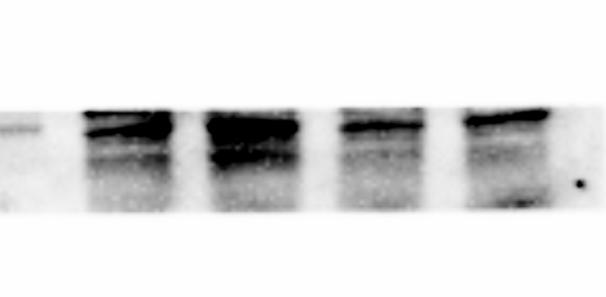


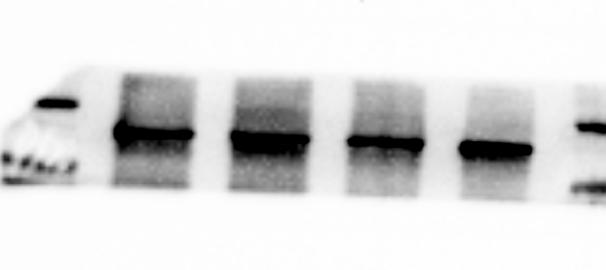

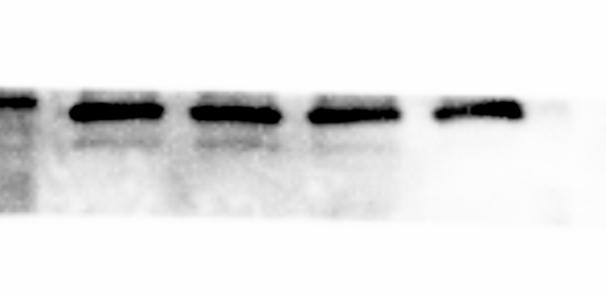


Fig. 7E


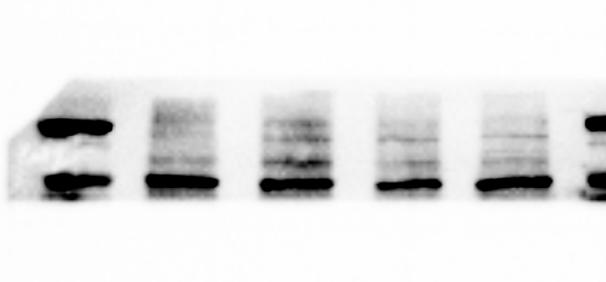


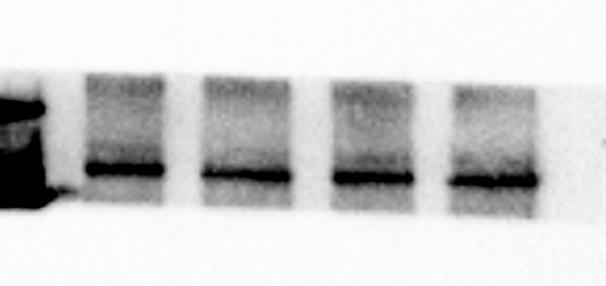


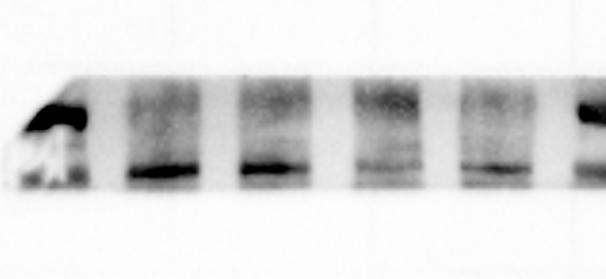


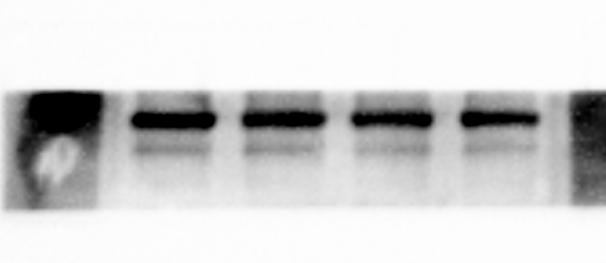

Supplement: Supplementary file 2 — Raw images of western blots [file 41419_2023_5790_MOESM2_ESM.docx]
